# Incongruity of Geometric and Spectral Markers in the Assessment of Body Sway

**DOI:** 10.3389/fneur.2022.929132

**Published:** 2022-07-18

**Authors:** Stefania Sozzi, Shashank Ghai, Marco Schieppati

**Affiliations:** ^1^Istituti Clinici Scientifici Maugeri IRCCS, Centro Studi Attività Motorie (CSAM), Pavia, Italy; ^2^Department of Physical Therapy, Rsgbiogen, New Delhi, India

**Keywords:** stance, CoP excursion, vision, support surface, adaptation, Sway Area, Path Length, frequency spectrum

## Abstract

Different measurements of body oscillations in the time or frequency domain are being employed as markers of gait and balance abnormalities. This study investigates basic relationships within and between geometric and spectral measures in a population of young adult subjects. Twenty healthy subjects stood with parallel feet on a force platform with and without a foam pad. Adaptation effects to prolonged stance were assessed by comparing the first and last of a series of eight successive trials. Centre of Foot Pressure (CoP) excursions were recorded with Eyes Closed (EC) and Open (EO) for 90s. Geometric measures (Sway Area, Path Length), standard deviation (SD) of the excursions, and spectral measure (mean power Spectrum Level and Median Frequency), along the medio-lateral (ML) and antero-posterior (AP) direction were computed. Sway Area was more strongly associated than Path Length with CoP SD and, consequently, with mean Spectrum Level for both ML and AP, and both visual and surface conditions. The squared-SD directly specified the mean power Spectrum Level of CoP excursions (ML and AP) in all conditions. Median Frequency was hardly related to Spectrum Level. Adaptation had a confounding effect, whereby equal values of Sway Area, Path Length, and Spectrum Level corresponded to different Median Frequency values. Mean Spectrum Level and SDs of the time series of CoP ML and AP excursions convey the same meaning and bear an acceptable correspondence with Sway Area values. Shifts in Median Frequency values represent important indications of neuromuscular control of stance and of the effects of vision, support conditions, and adaptation. The Romberg Quotient EC/EO for a given variable is contingent on the compliance of the base of support and adaptation, and different between Sway Area and Path Length, but similar between Sway Area and Spectrum Level (AP and ML). These measures must be taken with caution in clinical studies, and considered together in order to get a reliable indication of overall body sway, of modifications by sensory and standing condition, and of changes with ageing, medical conditions and rehabilitation treatment. However, distinct measures shed light on the discrete mechanisms and complex processes underpinning the maintenance of stance.

## Introduction

When one plots in a scattergraph the values of the length of the path travelled by the centre of feet pressure (CoP) of a population of subjects standing on a force plate against the values of the respective sway area, one wonders why the dots do not define a straight line or, at least, do not always change in the same direction. Beyond a broad proportional configuration, the cloud identified by the dots can be quite large and variable, and the value of the coefficient of determination of the linear regression may be weak. Why this is so is not obvious.

We have become recently interested in this, perhaps, vacuous issue by the observation that these two markers of body sway diverge in their values during the process of adaptation to repeated consecutive standing trials (1). When standing on foam without vision, Path Length decreased while Sway Area remained approximately constant or moderately increased over time. Conversely, the spectral analysis of the CoP time series in the anteroposterior (AP) and mediolateral (ML) directions showed that the total amplitude of the Spectrum remained almost constant with trial repetition as well, while the Median Frequency of the spectrum decreased. Specifically, with the adaptation to successive stance trials, the Spectrum Level increased in the low oscillation frequency range and decreased at high frequencies. This directly affected the value of the Median Frequency that moved towards lower values, whereas the total amplitude of the Spectrum could remain unaltered or increased.

Physiologically, this would mean that rapid postural adjustments superimposed onto slower body oscillations tended to disappear within a sequence of trials, thereby decreasing Path Length. However, the area of the surface covered by the wandering CoP (Sway Area) would increase because the CoP, not any longer restrained by a “stiffening strategy” (2, 3), moved across wider distances with smoother excursions characterised by mainly low frequencies (4). Our findings were interpreted as a modification in the postural control from a prevalent trembling to a prevalent rambling behaviour (5–7).

The relevance of the issue goes well beyond the investigation of the understudied mechanisms contributing to the process of adaptation of standing balance (1, 8–10), and covers other areas. Whether or not adaptation can be a model for addressing balance problems in clinical populations, one can often encounter largely diverging measures of Path Length (or velocity) and Sway Area across populations of people of different ages (11–13) or with balance problems, independently of the adaptation process.

Several investigations have proposed postural sway measures that could become markers of age effect on postural control or of increased fall risk in healthy ageing and in disease conditions [e.g., (14–19)]. On a different vein, it has been suggested that sway may not be a reliable marker of functional balance performance (20), and it seems that traditional measures of postural sway are not always able to differentiate between healthy subjects and patients with movement disorders (21, 22). These uncertainties are partly dependent on technical or practical reasons, such as the recording apparatus, the duration of the acquisition period (23, 24), the frequency of sampling of the CoP excursions, the filtering procedures, and other confounding subject-related circumstances such as fatigue (25), not to speak of the instruction given to the participants and the ample inter-individual variability (17, 26–34).

On the theoretical side, there is still little consensus in the literature regarding the mechanisms underlying postural control (35), which is sometimes considered in the clinical practice as a motor function separate from the general human motility, or a task simply based on mechanical muscle properties or sensory feedback (36). CoP recordings have sometimes represented a field to exercise analytical virtuosities in the expectation that, from perfect knowledge of its variations during stance on a solid surface, one could derive general principles applicable to the control of human balance. In this connexion, one may simply note the limitations of the inverted pendulum model or of the supposed predominant role of the calf muscles (either stiffening the ankle joint or being activated by spinal reflexes) (37, 38). Only recently, the complex links between body segments and the interactions of the lower levels of the spinal cord and the higher brain centres in the control of posture have been addressed and brought to light (39–42).

However, a very basic issue, such as the pattern of the CoP excursion and the relationship between Sway Area and Path Length, might still merit to be addressed, to at least recognise potential sources of variability, given the importance attributed to these measures in estimating the quality of postural control. Sway Area (the surface of the 95% confidence ellipse fitted to the sampled ML and AP data) refers to the overall postural sway (43), while Path Length provides information about the pattern of the CoP excursions (44) when the recording period is constant across recorded trials and when equal periods of time are compared across participants. Path Length has been considered to possess a greater sensitivity over Sway Area for detecting changes in body sway (45, 46) and to be a predictor of falls (47, 48). Other study showed that balance training did not modify Sway Area but diminished Path Length on foam (49). Delmas et al. (50) have reported recently that the Standard Deviation (SD) of the anteroposterior excursion of the CoP is able to distinguish the postural control between young and old adults. The considerations above would encourage an unconventional reappraisal.

Building on our prior works (1, 51), we have hypothesised that a parallel analysis of geometric and spectral markers of the CoP oscillations would yield additional information about the standing strategy (8, 52–54), and hopefully help the interpretation of both geometric and spectral findings in order to better define postural control markers in healthy and diseased persons. Furthermore, we expected that this information would help understand how adaptation interacts with the operation of the neural mechanisms controlling stance. Therefore, this communication is centred on the study of the basic relationships within and between geometric and spectral data in a population of young adult subjects. Data from different sensory conditions (by manipulating vision and a support surface) and the changes over time of body sway with adaptation have been analysed in order to increase data variability and enhance the interpretation of the comparison between geometric and spectral markers.

## Methods

### Participants

Twenty healthy young adults (9 men and 11 women) participated in this study. Age was 29 ± 4.4 years (mean ± SD); height, 171.9 ± 6.3 cm; and weight, 67 ± 12.1 kg. The participants were resident physicians or physiotherapists at the Istituti Clinici Scientifici Maugeri SB, Pavia, Italy. Data had been recorded in a previously published study (1). Two subjects were removed because stance on Solid support was not completed. All the participants were in good condition, had no sight problems or their visual acuity was corrected, were free of neurological and musculo-skeletal disorders, and none had had vertigo episodes in the past. No subject reported injuries or occurrences of falls in the previous year. Participation was voluntary, written consent was obtained as conformed to the Declaration of Helsinki, and the protocol was approved by the local review board (Istituti Clinici Scientifici Maugeri SB, approval No. #2564-CE).

### Procedures

The subjects stood barefoot, for at least 100 s on a force platform (Kistler 9286BA, Switzerland), with the outer profiles of the parallel feet at hip width. This will be referred to as the Solid surface condition. For the Foam condition study, a foam pad (Airex Balance Pad, Switzerland; L 50 cm; W, 41 cm; H, 6 cm; density, 55 g/dm^3^; Young's module, 260 kP) was placed onto the platform (55). The feet position was marked on a paper sheet placed on top of the platform or foam pad for consistency across trials. Under both Solid and Foam surfaces, balance was measured with eyes open (EO) and with eyes closed (EC). The subjects stood at ease (28, 56, 57) and looked at the structured visual scene of the laboratory wall at 6-m distance (51, 58). All the subjects were naive to foam standing.

Each volunteer came to the laboratory on separate four days in order to record “adapted” trials in the diverse conditions (EO and EC, each on the Solid surface and Foam). These were the last ones of a series of eight consecutive trials under the same sensory condition, subsequently performed in the same experimental session. There were no preliminary practice trials. The last 90-s-epoch of each 100-s stance trial was acquired (59) in order to exclude the adjusting phase occurring after stepping onto the foam pad. There were short intervals between the trials (15- to 30-s long), when the subjects freely made a few steps. When asked at the end of the trial sequence, the subjects reported no fatigue. None of the subjects lost balance whilst standing on Foam despite the increase in sway compared to the Solid support surface.

### Data Acquisition and Processing

Details about data acquisition and processing are reported in Sozzi et al. (51). Briefly, the platform data from which the CoP was calculated were acquired at the sampling frequency of 140 Hz by dedicated software (Smart-D, BTS, Italy). A post-acquisition analysis was done using Excel (Microsoft), MATLAB (MathWorks) and LabVIEW (National Instrument). The force platform signals of the CoP excursions along the anteroposterior (AP) and mediolateral (ML) directions were high-pass filtered at 0.01 Hz and low-pass filtered at 20 Hz, with a 4^th^-order Butterworth filter, after removing the respective mean values. The length of the path (Path Length) was the total length of the time series (90 s) of the wandering CoP. Sway Area was the surface of the 95% ellipse fitted to the dispersion of the time-series data on the horizontal plane (60). As a common index of sway, the Standard Deviation (SD) of the CoP oscillations along both the ML and the AP directions was also calculated.

The frequency analysis was performed by means of the Auto power spectrum Virtual Instrument (VI) algorithm of the LabVIEW functions. This VI calculated the fast Fourier transform of the CoP ML and AP time-series of each trial, subject, visual, and support surface condition. The VI produced a single-sided power spectrum (the positive half of the frequency spectrum from 0.01 to 70 Hz, corresponding to a length of 6,300 points). The resolution (sampling frequency/sample number of the CoP signal) was 0.011 Hz for the sampling frequency of 140 Hz (51, 61). The power spectrum signal was expressed in ${\text{cm}}_{\text{rms}}^{2}$. The subscript root mean square (rms) is omitted in the figures. Of note, the rms of a zeroed CoP signal is equal to the CoP standard deviation (AP and ML), so the rms^2^ is equal to the variance of the signal (the squared SD in the following text). We have analysed the power spectrum from 0.01 to 2.0 Hz, i.e., in the frequency range containing 98 % of the power of a spectrum frequency up to 70 Hz. In this range, the number of samples of the spectrum is 180. The number of the spectrum points affects the slope of the relationship between the mean amplitude of the CoP power spectrum and the CoP squared SD (see Results).

Hence, for each subject, trial, and conditions, we calculated the Median Frequency and the mean Spectrum Level (the arithmetic mean of all amplitude values for each sampled frequency) in the range 0.01 to 2.0 Hz. These variables were calculated for both the ML and the AP directions. The Romberg Quotient (RQ, EC/EO) in the Foam and Solid support was calculated for the Sway Area, Path Length, mean Spectrum Level and Median Frequency for both ML and AP directions. The same analytical procedures were applied to the first (non-adapted) and the last trials (adapted) of each subject in each of the four conditions tested.

### Data Treatment and Statistics

The SD of the CoP excursions along the ML and AP directions, Path Length and Sway Area, Median Frequency, and mean Spectrum Level was considered. The relationship between the geometric measures was studied by a linear regression model, and the coefficient of determination (*R*^2^) was calculated. For each subject and condition, the Sway Area was plotted against the corresponding value of CoP Path Length and of the ML and AP squared SD. Path length was plotted against ML and AP squared SD. The same procedure was used to study the relationship between the mean Spectrum Level and the Median Frequency for both the ML and AP directions. CoP ML and AP SD and squared SD were plotted against the mean Spectrum Level. The regression lines (the slope and the intercept) were compared between conditions by means of the Compare Linear Fit Parameters routine of the software Origin® (OriginLab Corporation, Northampton, MA, USA). The RQs EC/EO of the mean AP spectrum level were plotted against the RQs calculated for the Sway Area for both Foam and Solid conditions. The RQs of the AP Median Frequency were instead plotted against the RQs of the CoP Path Length for both Foam and Solid conditions. In both cases, a linear regression model fit the data of Foam and Solid conditions.

Assumptions for parametric statistics, as assessed by the Kolmogorov-Smirnov and Levene's test, were met for all the variables of interest, except for the RQs. The effects of the different visual and base of support conditions on Path Length and Sway Area were compared by 2-(Solid or Foam)-x-2 (visual conditions) repeated measures (rm) ANOVA. The ML and AP SDs and the mean level of power spectrum were compared by a 2- (ML or AP direction)-x-2-(Solid or Foam)-x-2 (EO or EC) rm ANOVA. The comparisons of the geometric and spectral measures between the first (non-adapted) and the last (adapted) trials were also performed. A 2- (non-adapted or adapted trial)-x-2-(Solid or Foam)-x-2 (visual conditions) rm ANOVA was used to compare Sway Area and Path Length. A 2-(non-adapted or adapted trial)-x-2- (ML or AP directions)-x-2 (Solid or Foam)-x-2 (visual conditions) rm ANOVA was used to compare: SDs, mean Spectrum Levels and Median Frequencies. Where the differences were significant, the $\eta_{\text{p}}^{2}$ was reported. The *post-hoc* test was the Fisher's LSD test. The Romberg Quotients (RQ) were compared by the Friedman's non-parametric ANOVA. The *post-hoc* Wilcoxon's test (with the Bonferroni correction for the multiple comparisons) was performed when a significant difference was detected. The minimum effect size given our sample size (*n* = 20) was calculated using G^*^Power (62). With this sample, the study proved to have a sufficient power (>80%) to detect an effect size (Cohen's *d*) of 0.57. Statistical tests were performed using Statistica (Statsoft, USA). The significance level was always set at *p* < 0.05.

## Results

The findings will be described in two parts; the former (subheadings 1st to 5th) reports the results of the analysis of the sway variables in the non-adapted standing trials, the latter (6th and 7th) in the adapted trials.

### Sway Area and Path Length and Their Reciprocal Relationship in the Non-adapted Trials

**Figure 1:**
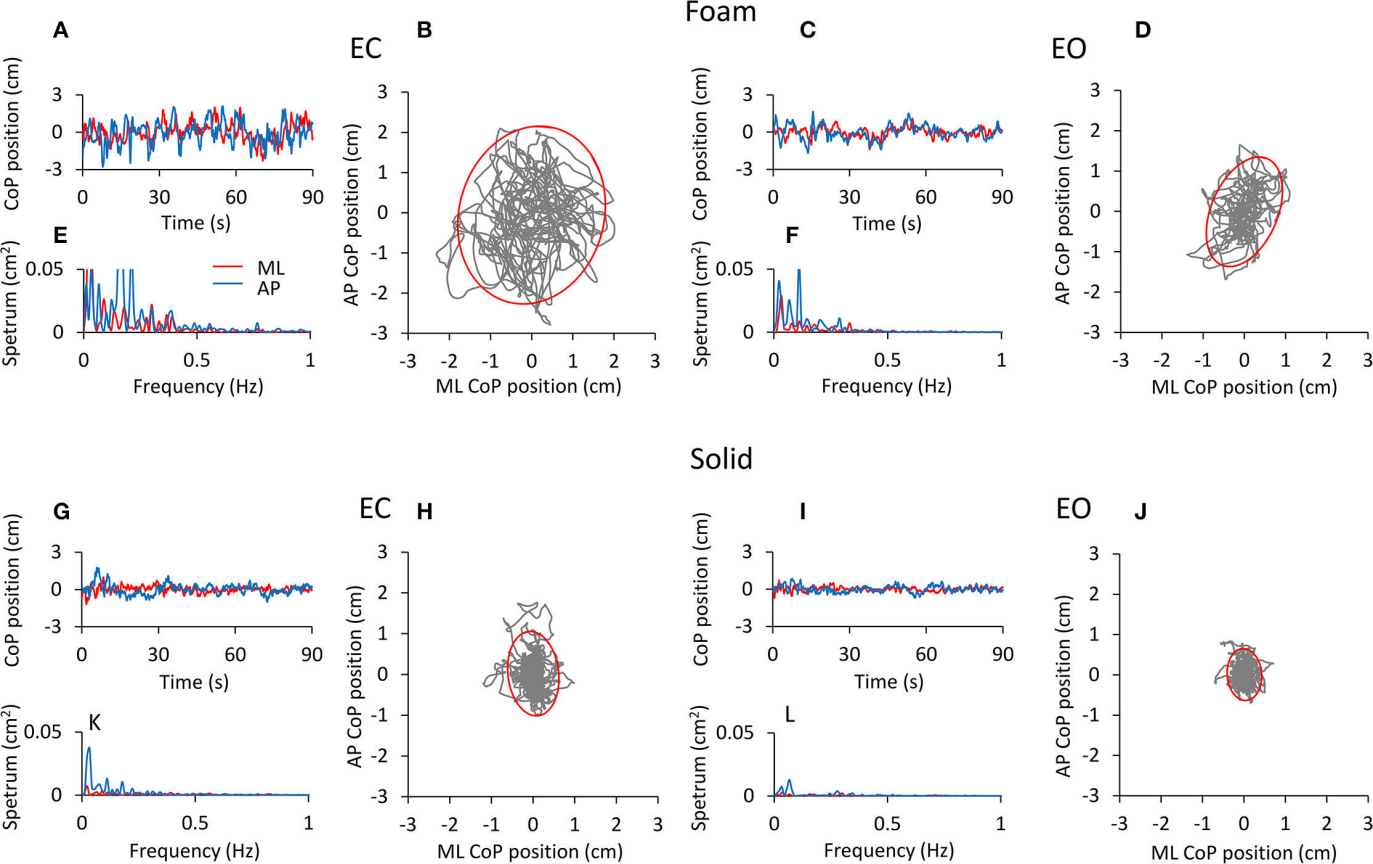
CoP excursion and power spectrum in a representative subject (top panels, Foam; bottom panels, Solid). The CoP excursions along the ML (red traces) and AP (blue traces) directions are reported for the four conditions of interest [**(A)**, EC Foam; **(C)**, EO Foam; **(G)**, EC Solid; **(I)**, EO Solid]. The CoP excursions in the horizontal plane and the 95% prediction ellipses are shown for EC **(B,H)** and EO **(D,J)** conditions, both with Foam **(B,D)** and Solid **(H,J)** supports. The corresponding power spectra are reported in **(E,F,K,L)** for both ML (red) and AP (blue) directions.

Figure 1 shows examples of recordings of the CoP excursions in the mediolateral (ML, red traces) and in the anteroposterior (AP, blue traces) directions (Figures 1A,C,G,I), the frequency of spectra (Figures 1E,F,K,L) of these excursions and the trajectory of the CoP in the horizontal plane (Figures 1B,D,H,J) for a representative subject standing in the four experimental conditions tested. The subject stood on the Foam (upper panels) and on the Solid support (lower panels). The 95% prediction ellipses (red) are superimposed on the corresponding CoP excursions. Their area represents here the surface within which the body sway takes place.

The CoP excursions were broadly omnidirectional for both Foam and Solid supports, with a minor dominance for the AP direction. They were smaller with EO than EC under both surface conditions, and were much smaller under both visual conditions when the subject stood on the Solid than on the Foam surface. Also, spectrum amplitudes were greater with Foam (Figures 1E,F) than the Solid (Figures 1K,L) support surface. Spectrum amplitudes were reduced with vision on both the AP and the ML directions. The oscillation frequencies were comprised in a broader range on Foam than the Solid surface (particularly for EC), and their spectrum had a smaller amplitude with Solid than Foam support and with EO than EC. The largest peaks were in the lowest frequency ranges and were definitely higher in the AP than ML direction.

**Figure 2:**
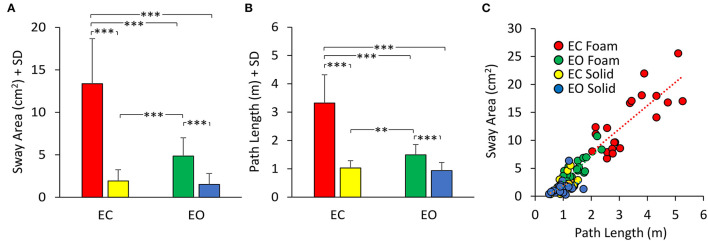
Mean Sway Area **(A)** and Path Length **(B)**. Sway Area and Path Length are greater in Foam (EC, red; EO, green) than in Solid support condition (EC, yellow; EO, blue). Vision has a greater effect with Foam than Solid support for both Sway Area and Path Length. **(C)** the relationship between Sway Area and Path Length is shown for all visual and support conditions. Each dot corresponds to a subject. Asterisks indicate significant differences (**p < 0.01, ***p < 0.001).

Figure 2 shows the mean values (average of all subjects) of Sway Area (Figure 2A) and Path Length (Figure 2B) in the four experimental conditions, and the relationship between Sway Area and Path Length (Figure 2C). The height of the bars was smaller for both Sway Area (Figure 2A) and Path Length (Figure 2B) in the Solid (yellow and blue bars) compared to the Foam support (red and green bars). For the Foam support, the values of the Sway Area and Path Length were different between visual conditions with respect to the Solid support, where vision had little effect. On the whole, Sway Area and Path Length were greater under Foam than the Solid support surface [main effect of support; Sway Area, *F*_(1, 19)_ = 149.06, *p* < 0.001, $\eta_{\text{p}}^{2}$ = 0.88; Path Length, *F*_(1, 19)_ = 158.98, *p* < 0.001, $\eta_{\text{p}}^{2}$ = 0.89]. Vision had effects as well (EO < EC) [main effect of vision; Sway Area, *F*_(1, 19)_ = 93.78, *p* < 0.001, $\eta_{\text{p}}^{2}$ = 0.83; Path Length, *F*_(1, 19)_ = 90.14, *p* < 0.001, $\eta_{\text{p}}^{2}$ = 0.83]. The interaction between support surface and visual conditions was also significant [Sway Area, *F*_(1, 19)_ = 86.04, *p* < 0.001, $\eta_{\text{p}}^{2}$ = 0.82; Path Length, *F*_(1, 19)_ = 83.01, *p* < 0.001, $\eta_{\text{p}}^{2}$ = 0.82]. For the Foam support, the values of Sway Area and Path Length were both remarkably different between visual conditions (*post-hoc, p* < 0.001 for both Sway Area and Path Length) with respect to the Solid support, where vision had instead little effect (*post-hoc*, Sway Area, *p* = 0.9; Path Length, *p* = 0.88). With EC Foam, Sway Area and Path Length were the greatest with respect to other visual and base of support conditions (*p* < 0.001, for all comparisons). With EO Foam, Sway Area and Path Length were greater than in Solid condition with both EC and EO (*p* < 0.05 for all comparisons).

In Figure 2C, the dots represent the values of the individual subjects, where the colours indicate the support type and the visual condition (blue and yellow refer to Solid; green and red to Foam). The dots are clustered towards low Sway Area and Path Length values for the Solid condition whilst the greater data values (for Foam) occurred on the right upper part of the graph. With Foam, the values of both Sway Area and Path Length variables were relatively small with vision (EO, green), whereas closing the eyes (EC) moved the dots to the right and upwards. Table 1 shows the equations of the best fit lines for the four conditions, their coefficients of determination (*R*^2^) and the probabilities for the slopes to be significantly different from zero. It also indicates which slopes were different from each other. The relationships were steeper with Foam than Solid, indicating proportionally larger values for Sway Area than Path length on Foam than on Solid support. The slopes pertaining to EC and EO conditions with Foam were similar. The determination coefficients were greater with Foam than Solid support, where they were small and barely or not significant.

**Table 1:** Relationship between Sway Area and Path Length.

**Condition**	**Equation**	***R^**2**^***	**Slope different from zero**	**Difference between conditions**
EC foam	y = 4.18 x−0.51	0.61	*p* < 0.001	EC foam vs. EO foam: *p* = 0.82 EC foam vs. EC solid: *p* = 0.34 EC foam vs. EO solid: *p* = 0.16
EO foam	y = 4.47 x−1.8	0.58	*p* < 0.001	EO foam vs. EC foam: *p* = 0.82 EO foam vs. EC solid: *p* = 0.06 EO foam vs. EO solid: *p* < 0.01
EC solid	y = 2.77 x−0.93	0.29	*p* < 0.05	EC solid vs. EC foam: *p* = 0.34 EC solid vs. EO foam: *p* = 0.06 EC solid vs. EO solid: *p* = 0.59
EO solid	y = 1.53 x + 0.08	0.12	*p* = 0.14	EO solid vs. EC foam: *p* = 0.16 EO solid vs. EO foam: *p* < 0.01 EO solid vs. EC solid: *p* = 0.59

### Standard Deviation of the CoP Excursions and Mean Spectrum Level in the AP and ML Directions and Their Relationship in the Non-adapted Trials

**Figure 3:**
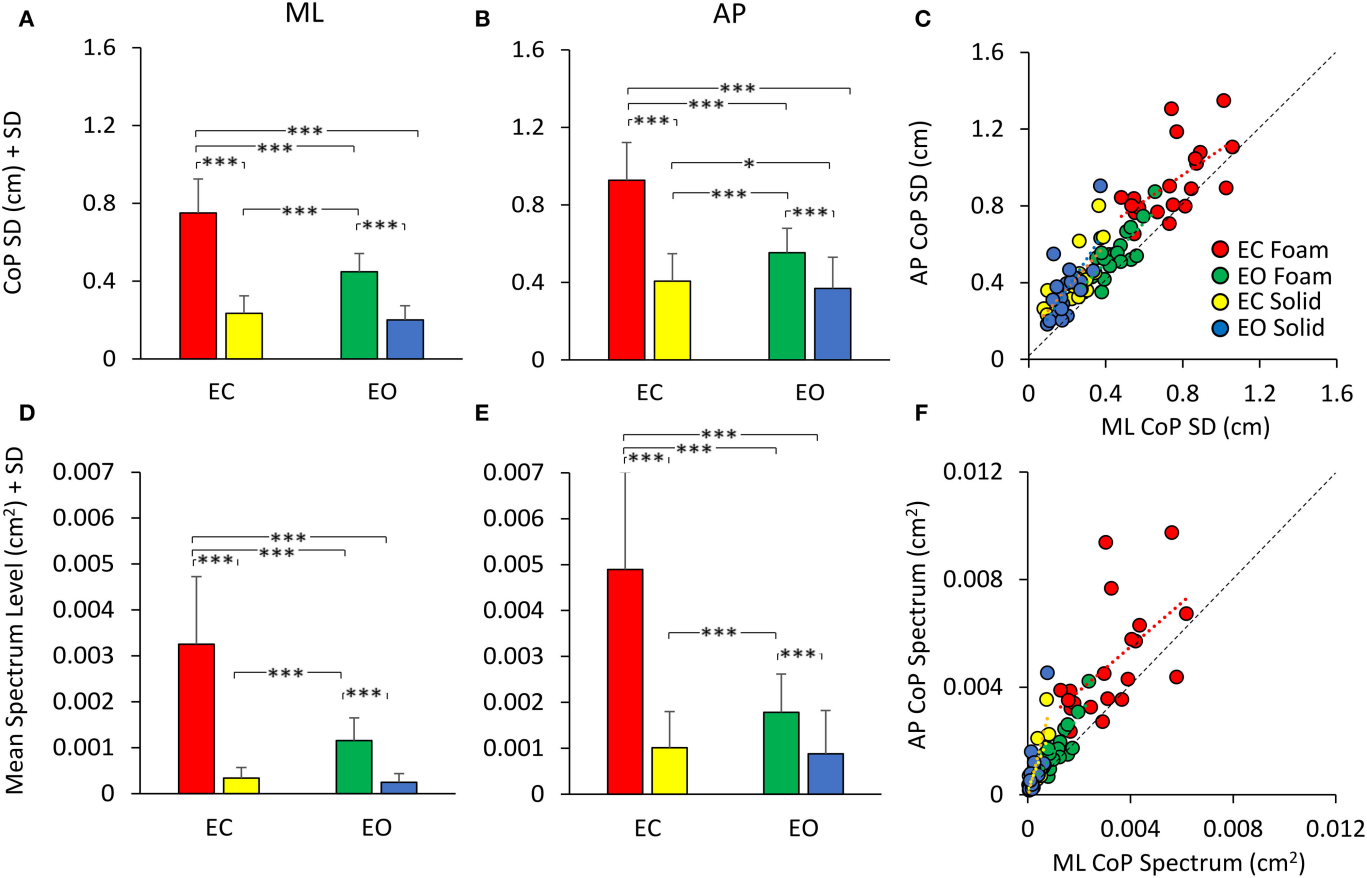
CoP SD (top panels) and mean Spectrum Level (bottom panels). The mean CoP SDs across subjects are reported for the ML **(A)** and AP **(B)** directions for all visual (EC, red and yellow; EO green and blue) and support (Foam, red and green; Solid, yellow and blue) conditions. The mean Spectrum Level is reported for both ML **(D)** and AP **(E)** directions [same colour code as in **(A,B)**]. The CoP SD and mean Spectrum Level are greater with Foam than Solid support. Vision removal affects SD and Spectrum Level mainly with Foam support (red and yellow). **(C)** shows the relationship between AP and ML SDs. Both CoP SDs increased with a broadly similar pattern from the more- to the less-stable conditions. **(F)** shows the relationship between AP and ML mean Spectrum Level. Much as for the SDs, there was a proportionality between AP and ML Spectrum Level. Each dot in C and F corresponds to a subject. Asterisks indicate significant differences (*, *p* < 0.05; ***, *p* < 0.001).

The mean values of the standard deviation (SD) of the CoP excursion in ML and AP directions are reported in Figures 3A,B. The similarity with the graphs of Sway Area and Path Length (compare with Figure 2) is evident. However, the representation of the SDs underlines the properties of the body sway in the ML and AP directions, which cannot be obtained solely by Sway Area and Path Length. Here, the difference in the SDs was significant (AP > ML) [main effect, *F*_(1, 19)_ = 82.58, *p* < 0.001, $\eta_{\text{p}}^{2}$ = 0.81], as was the difference between the support surface conditions (Foam > Solid) [*F*_(1, 19)_ = 389.6, *p* < 0.001, $\eta_{\text{p}}^{2}$ = 0.95] and visual conditions (EC > EO) [main effect, *F*_(1, 19)_ = 151.3, *p* < 0.001, $\eta_{\text{p}}^{2}$ = 0.89]. With EC Foam, the SD was the greatest for both ML and AP directions (*post-hoc, p* < 0.001 for all comparisons). The difference between visual conditions with the Solid support was small in ML direction (*p* = 0.08), whilst it reached significance in AP direction (*p* < 0.05).

Figure 3C shows the relationship between the SDs in the AP and ML directions. Across the population, under all support and visual conditions, both SDs increased with a broadly similar pattern. The regression line through the pooled dots was parallel to the identity line, and the intercept was different from zero (*t*-test, *p* < 0.001), confirming that the SD values were overall larger in AP than in ML direction. Table 2 shows the equations of the four best fit lines, their determination coefficients, and the probabilities of the slopes to be significantly different from zero and different between conditions.

**Table 2:** Relationship between ML and AP SDs.

**Condition**	**Equation**	***R^**2**^***	**Slope different from zero**	**Difference between conditions**
EC foam	y = 0.67 x + 0.42	0.36	*p* < 0.01	EC foam vs. EO foam: *p* = 0.84 EC foam vs. EC solid*: p* = 0.75 EC foam vs. EO solid: *p* = 0.26
EO foam	y = 1.05 x + 0.08	0.61	*p* < 0.001	EO foam vs. EC foam: *p* = 0.84 EO foam vs. EC solid: *p* = 0.43 EO foam vs. EO solid: *p* < 0.05
EC solid	y = 1.15 x + 0.14	0.54	*p* < 0.001	EC solid vs. EC foam: *p* = 0.75 EC solid vs. EO foam: *p* = 0.43 EC solid vs. EO solid: *p* = 0.22
EO solid	y = 1.54 x + 0.06	0.48	*p* < 0.001	EO solid vs. EC foam: *p* = 0.26 EO solid vs. EO foam: *p* < 0.05 EO solid vs. EC solid: *p* = 0.22

In Figures 3D,E, the bars indicate the mean Spectrum Level between 0.01 and 2.0 Hz for both ML (Figure 3D) and AP (Figure 3E) directions, for both support surfaces (Foam, red and green; Solid, yellow and blue) and for both visual conditions (EC, red and yellow, and EO, green and blue). There was a difference between ML and AP directions (AP > ML) [main effect, *F*_(1, 19)_ = 163.89, *p* < 0.001, $\eta_{\text{p}}^{2}$ = 0.85], as observed for the SD. The spectrum was also larger with Foam than Solid support [main effect, *F*_(1, 19)_ = 106.6, *p* < 0.001, $\eta_{\text{p}}^{2}$ = 0.89] and with EC than EO [main effect, *F*_(1, 19)_ = 93.6, *p* < 0.001, $\eta_{\text{p}}^{2}$ = 0.83]. In the EC Foam condition, the mean Spectrum Level was the greatest compared to all other conditions both in ML and AP directions (*p* < 0.001 for both comparisons). With the Solid support, there was no difference between visual conditions in both ML and AP directions (*p* > 0.47 for all comparisons). The comparison of the pooled dots of the scatterplot with the identity line confirmed that the SD values were overall larger in AP than in ML direction. The regression line was parallel to the identity line, and the intercept was different from zero (*t*-test, *p* < 0.001). Therefore, there was a substantial similarity between SDs and spectra (Figure 3F), which held both for vision and for support conditions. Across the participants, there was a similar proportionality between SD and the spectrum in both the ML and AP directions, except for a larger scatter of the AP dots in the EC Foam condition.

### The Mean Level of the Spectrum Is Determined by the Variance of the CoP Excursions

**Figure 4:**
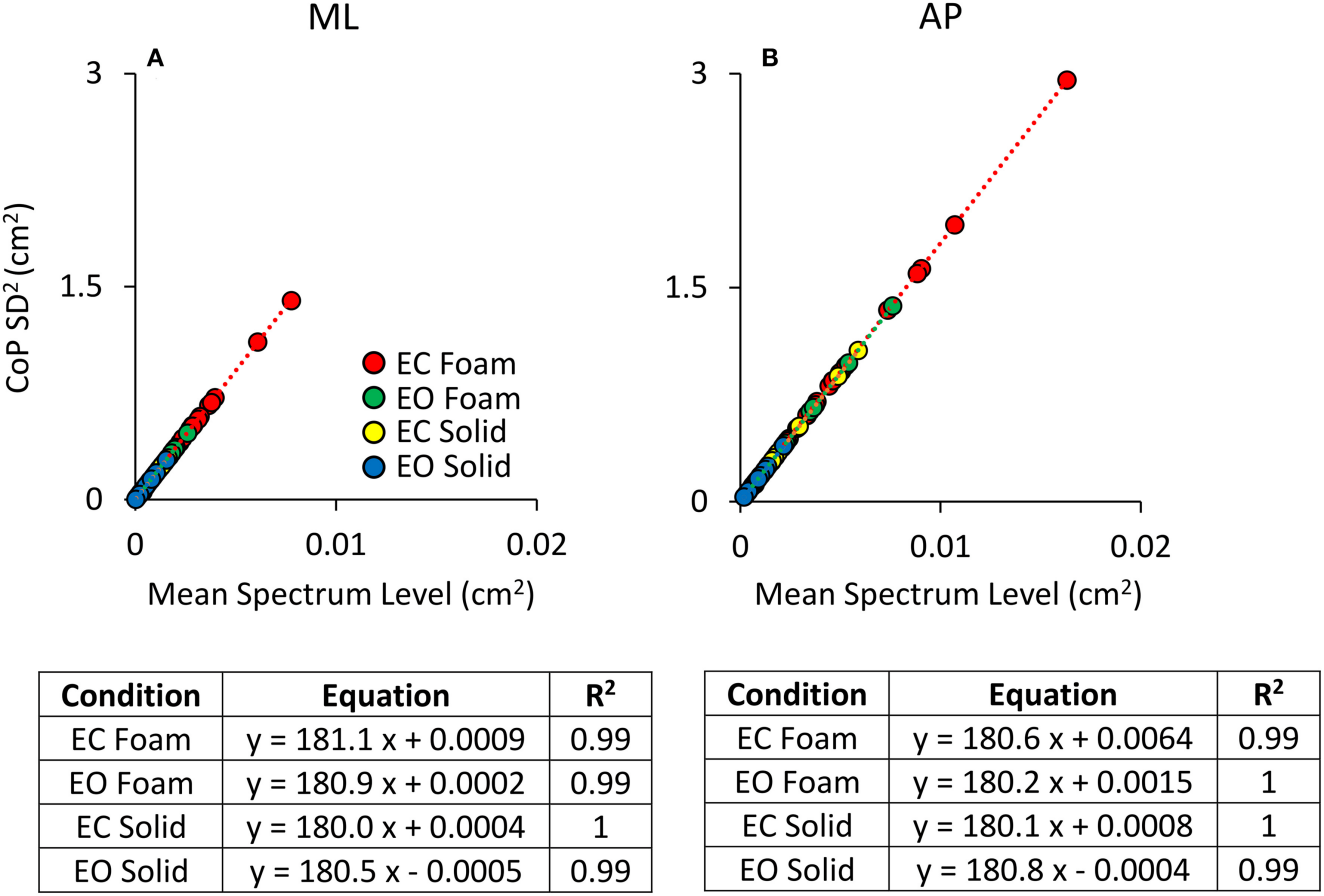
Relationship between CoP variance (SD^2^) and Spectrum Level. The values of the CoP SD^2^ are plotted against the corresponding values of the mean Spectrum Level for both ML **(A)** and AP **(B)** directions and both visual and surface conditions (EC Foam, red; EC Solid, yellow; EO Foam, green; EO Solid, blue). Each dot corresponds to a subject. The slope of the lines best fitting the relationship between CoP SD^2^ and mean Spectrum Level is identical between visual and support conditions and between ML and AP directions.

Figure 4 shows that the relationship between the squared SD and the mean Spectrum Level is a straight line. Figure 4A refers to ML; Figure 4B to AP direction. The line best fitting the relationship of CoP squared SD (SD^2^ in the graphs) and mean Spectrum Level is identical for Solid and Foam support, for both visual conditions and for both ML and AP directions (see the tables included in Figure 4). Hence, the squared SD directly determines the value of the power spectrum of the time series of the CoP excursions along a given direction (ML or AP). According to the formula for the calculation of the Spectrum Level, the slope of the line corresponds to the number of the frequency samples comprised in the 0.01–2.0 Hz interval (i.e., 180 points).

Regardless of the absolute values of the squared SD in the ordinates and the different vision and surface conditions (the four colours), the regression coefficients of the four straight lines fitted to the data of the four conditions are indistinguishable, and all determination coefficients are virtually 1. Same is true for both directions of the CoP excursions. In some cases, the slope of the regression line is not exactly 180 (ranging from 180.5 to 182.1). This is due both to the decimal approximations of the values of the mean Spectrum Level and of the SDs and to our decision to compute Spectrum Level up to 2.0 Hz, covering <100% of the full spectrum (98% of it was included), thereby possibly missing some higher-frequency excursion in some subjects. These necessary relationships indicate the equivalence of the CoP variance with the Spectrum Level across all possible standing conditions.

### Sway Area Bears a Stronger Association Than Path Length With the Variance of the CoP Excursions

**Figure 5:**
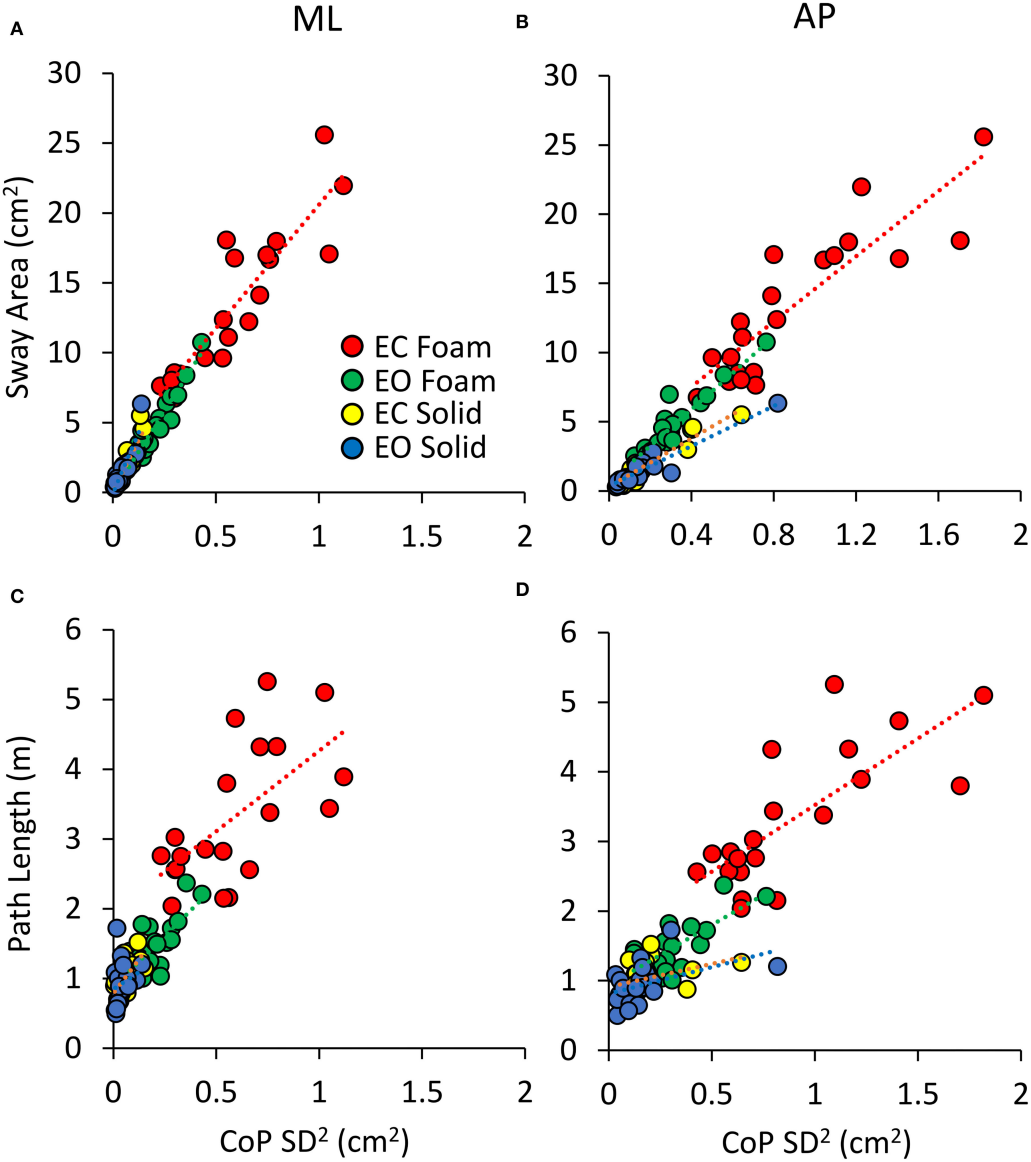
Relationship between geometric measures and variance of the CoP excursions. Sway Area **(A,B)** and Path Length **(C,D)** are plotted against the CoP variance (SD^2^) for both ML **(A,C)** and AP directions **(B,D)** for all visual and support conditions. Each dot corresponds to a subject. The association with CoPSD^2^ is stronger for Sway Area than for Path Length.

Figure 5 shows the relationship of Sway Area (Figures 5A,B) and Path Length (Figures 5C,D) with the variance (the squared SD) of the CoP excursions in ML and AP directions across all the subjects. Because of the mathematical relationship between squared SD and the power spectrum, this step is instrumental to address the correspondence between the geometric measures and the power spectrum of the time series of the CoP excursions. Tables 3, 4 show the equations of the lines fitting the values of Sway Area or Path Length, respectively, plotted against the ML or AP squared SD, their coefficients of determination, the probabilities for the slopes to be different from zero and different between conditions. The coefficients of determination were all greater for Sway Area than Path Length, indicating that the association with squared SD (or mean Spectrum Level) was stronger for Sway Area than Path Length, for both ML and AP directions, and for both visual and both surface conditions.

**Table 3:** Relationship between Sway Area and CoP SD^2^.

**Direction**	**Condition**	**Equation**	**R^**2**^**	**Slope different from zero**	**Difference between conditions**
ML	EC foam	y = 17.69 x + 2.9	0.80	*p* < 0.001	EC foam vs. EO foam: *p* = 0.26 EC foam vs. EC solid: *p* = 0.24 EC foam vs. EO solid: *p* = 0.16
	EO foam	y = 23.16 x + 0.004	0.92	*p* < 0.001	EO foam vs. EC foam: *p* = 0.24 EO foam vs. EC solid: *p* = 0.09 EO foam vs. EO solid: *p* = 0.25
	EC solid	y = 29.33 x + 0.09	0.84	*p* < 0.001	EC solid vs. EC foam: *p* = 0.24 EC solid vs. EO foam: *p* = 0.09 EC solid vs. EO solid: *p* = 0.25
	EO solid	y = 35.07 x - 0.08	0.82	*p* < 0.001	EO solid vs. EC foam: *p* = 0.16 EO solid vs. EO foam: *p* < 0.05 EO solid vs. EC solid: *p* = 0.25
AP	EC foam	y = 11.8 x + 2.8	0.76	*p* < 0.001	EC foam vs. EO foam: *p* = 0.66 EC foam vs. EC solid: *p* = 0.34 EC foam vs. EO solid: *p* = 0.11
	EO foam	y = 13.17 x + 0.61	0.86	*p* < 0.001	EO foam vs. EC foam: *p* = 0.66 EO foam vs. EC solid: *p* < 0.01 EO foam vs. EO solid: *p* < 0.001
	EC solid	y = 8.67 x + 0.33	0.87	*p* < 0.001	EC solid vs. EC foam: *p* = 0.34 EC solid vs. EO foam: *p* < 0.01 EC solid vs. EO solid: *p* = 0.14
	EO solid	y = 7.22 x + 0.34	0.89	*p* < 0.001	EO solid vs. EC foam: *p* = 0.11 EO solid vs. EO foam: *p* < 0.001 EO solid vs. EC solid: *p* = 0.14

**Table 4:** Relationship between Path Length and CoP SD^2^.

**Direction**	**Condition**	**Equation**	**R^**2**^**	**Slope different from zero**	**Difference between conditions**
ML	EC foam	y = 2.3 x + 1.96	0.39	*p* < 0.01	EC foam vs. EO foam: *p* = 0.70 EC foam vs. EC solid: *p* = 0.63 EC foam vs. EO solid: *p* = 0.94
	EO foam	y = 2.93 x + 0.87	0.51	*p* < 0.001	EO foam vs. EC foam: *p* = 0.70 EO foam vs. EC solid: *p* = 0.51 EO foam vs. EO solid: *p* = 0.64
	EC solid	y = 3.89 x + 0.79	0.39	*p* < 0.01	EC solid vs. EC foam: *p* = 0.63 EC solid vs. EO foam: *p* = 0.51 EC solid vs. EO solid: *p* = 0.39
	EO solid	y = 1.99 x - 0.85	0.05	*p* = 0.33	EO solid vs EC foam: *p* = 0.94 EO solid vs. EO foam: *p* = 0.64 EO solid vs. EC solid: *p* = 0.39
AP	EC foam	y = 1.92 x + 1.6	0.57	*p* < 0.001	EC foam vs. EO foam: *p* = 0.82 EC foam vs. EC solid: *p* = 0.15 EC foam vs. EO solid: *p* = 0.13
	EO foam	y = 1.72 x + 0.94	0.51	*p* < 0.001	EO foam vs. EC foam: *p* = 0.82 EO foam vs. EC solid: *p* = 0.07 EO foam vs. EO solid: *p* = 0.08
	EC solid	y = 0.66 x + 0.91	0.14	*p* = 0.106	EC solid vs. EC foam: *p* = 0.15 EC solid vs. EO foam: *p* = 0.07 EC solid vs. EO solid: *p* = 0.85
	EO solid	y = 0.77 x + 0.81	0.20	*p* < 0.05	EO solid vs. EC foam: *p* = 0.13 EO solid vs. EO foam: *p* = 0.08 EO solid vs. EC solid: *p* = 0.85

### Equal Values of Spectrum Level Correspond to Largely Different Median Frequency Values Across Participants and Conditions

**Figure 6:**
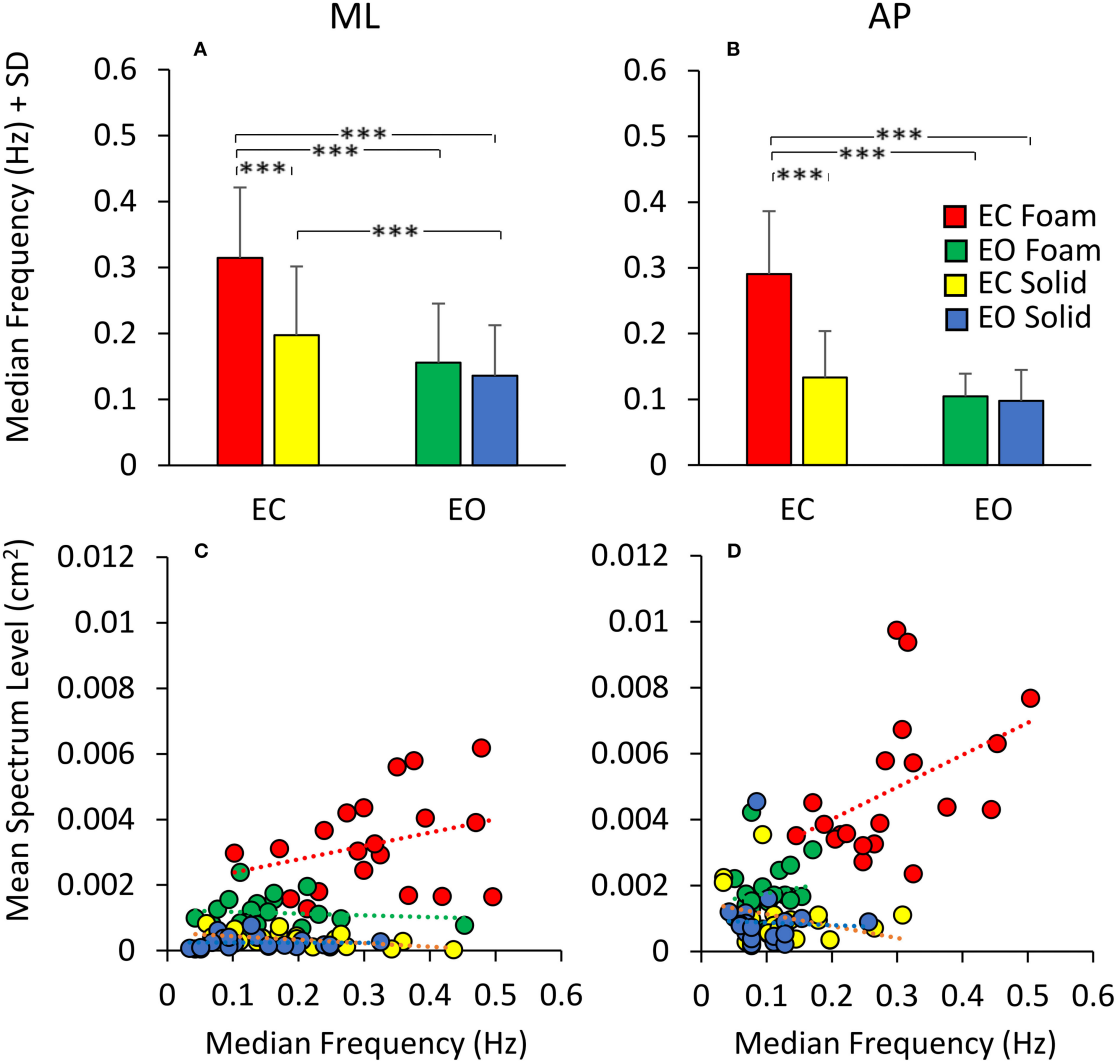
Median Frequency and Spectrum Level. The mean Median Frequency (average of all subjects) is reported for both ML **(A)** and AP **(B)** directions, both visual and both support conditions (red, EC Foam; green, EO Foam; yellow, EC Solid; blue, EO Solid). With Foam, the Median Frequency is higher than with Solid support for both ML and AP directions. With EO, the Median Frequency is smaller than that with EC, especially with Foam, for both ML and AP directions. The relationship between mean Spectrum Level and Median Frequency is reported in the lower panels for both ML **(C)** and AP **(D)** directions, both visual and both support conditions. Each dot corresponds to a subject. The Spectrum Level is not related to the value of the Median Frequency, both in ML and AP directions and with both visual and support conditions. Asterisks indicate significant differences (***p < 0.001).

The relationship of the spectrum amplitude with the CoP variance does not hold for the frequency data. In Figure 6, the mean values of the Median Frequency of the CoP time series for both visual and surface conditions and for both ML (Figure 6A) and AP (Figure 6B) directions are reported. The Median Frequency was higher on Foam than Solid support [main effect, *F*_(1, 19)_ = 27.5, *p* < 0.001, $\eta_{\text{p}}^{2}$ = 0.59], higher for EC than EO [main effect, *F*_(1, 19)_ = 93.2, *p* < 0.001, $\eta_{\text{p}}^{2}$ = 0.83], and higher for ML than AP [main effect, *F*_(1, 19)_ = 19.9, *p* < 0.001, $\eta_{\text{p}}^{2}$ = 0.51]. There was an interaction between Foam and Solid support and visual conditions [*F*_(1, 19)_ = 27.7, *p* < 0.001, $\eta_{\text{p}}^{2}$ = 0.59]. In the EC Foam condition, the Median Frequency was the greatest for both ML (*post-hoc, p* < 0.001 for all comparisons) and AP directions (*p* < 0.001 for all comparisons). Vision had no effect when standing on Solid support in AP direction (*post-hoc, p* = 0.16), whilst the Median Frequency in the ML direction was significantly greater with EC than EO (*p* = 0.02).

Figures 6C,D show that, across the subjects and regardless of the support and visual conditions, the Median Frequency of the spectrum featured values within a broad range, and the Spectrum Level was hardly related to the Median Frequency values. Whilst there was a clustering of the dots, with small mean levels of Spectrum Level associated with relatively lower Median Frequency values, in general, no trend for Spectrum Level vs. Median Frequency was obvious. The scatter of the Median Frequency values was grossly superimposable for EO Solid, EC Solid, and EO Foam (blue, yellow, and green dots).

The Spectrum Level generally showed increasing values as a function of ‘instability’ (EO Solid ~ EC Solid < EO Foam < EC Foam), but the regression lines for each of these conditions were not significantly different from zero (Table 5). For the EC Foam condition, both Median Frequency and Spectrum Level of the most subjects reached relatively high values, particularly in the AP direction, and the scatter across subjects was large. For instance, in the AP direction, six subjects had a similar Median Frequency around 0.3 Hz, with mean Spectrum Level ranging from 0.002 to 0.01 cm^2^. Conversely, for a mean spectrum level of about 0.004 cm^2^, the Median Frequencies varied between about 0.15 and 0.5 Hz.

**Table 5:** Relationship between mean Spectrum Level and Median Frequency.

**Direction**	**Condition**	**Equation**	**R^**2**^**	**Slope different from zero**	**Difference between conditions**
ML	EC foam	y = 0.004 x + 0.002	0.09	*p* = 0.2	EC foam vs. EO foam: *p* = 0.21 EC foam vs. EC solid: *p* = 0.11 EC foam vs. EO solid: *p* = 0.28
	EO foam	y = −0.0005 x + 0.001	0.009	*p* = 0.6	EO foam vs. EC foam: *p* = 0.70 EO foam vs. EC solid: *p* = 0.51 EO foam vs. EO solid: *p* = 0.64
	EC solid	y = −0.001 x + 0.0006	0.24	*p* < 0.05	EC solid vs. EC foam: *p* = 0.11 EC solid vs. EO foam: *p* = 0.69 EC solid vs. EO solid: *p* = 0.17
	EO solid	y = −0.00005 x + 0.0003	0.00004	*p* = 0.93	EO solid vs. EC foam: *p* = 0.28 EO solid vs. EO foam: *p* = 0.74 EO solid vs. EC solid: *p* = 0.17
AP	EC foam	y = 0.009 x + 0.002	0.19	*p* = 0.05	EC foam vs. EO foam: *p* = 0.55 EC foam vs. EC solid: *p* < 0.05 EC foam vs. EO solid: *p* = 0.21
	EO foam	y = 0.003 x + 0.0014	0.02	*p* = 0.56	EO foam vs. EC foam: *p* = 0.55 EO foam vs. EC solid: *p* = 0.25 EO foam vs. EO solid: *p* = 0.58
	EC solid	y = −0.004 x + 0.0015	0.10	*p* = 0.16	EC solid vs. EC foam: *p* < 0.05 EC solid vs. EO foam: *p* = 0.25 EC solid vs. EO solid: *p* = 0.58
	EO solid	y = −0.0008 x + 0.001	0.001	*p* = 0.87	EO solid vs. EC foam: *p* = 0.21 EO solid vs. EO foam: *p* = 0.58 EO solid vs. EC solid: *p* = 0.58

Table 5 shows the equations of the lines fitting the values of mean Spectrum Level plotted against the Median Frequency for both ML and AP directions, their regression coefficients, and the probabilities that the slopes were significantly different from zero and between conditions. Overall, this analysis showed that the relationship between Spectrum Level and Median Frequency was absent under “stabilised” conditions or modest and quite variable when standing on Foam with EC (in EO Foam, EC Solid, and EO Solid, the slope of the regression line was negative).

### The Effect of Adaptation on Geometric and Spectral Measures. Equal Values of Spectrum Level Correspond to Different Median Frequency Values

**Figure 7:**
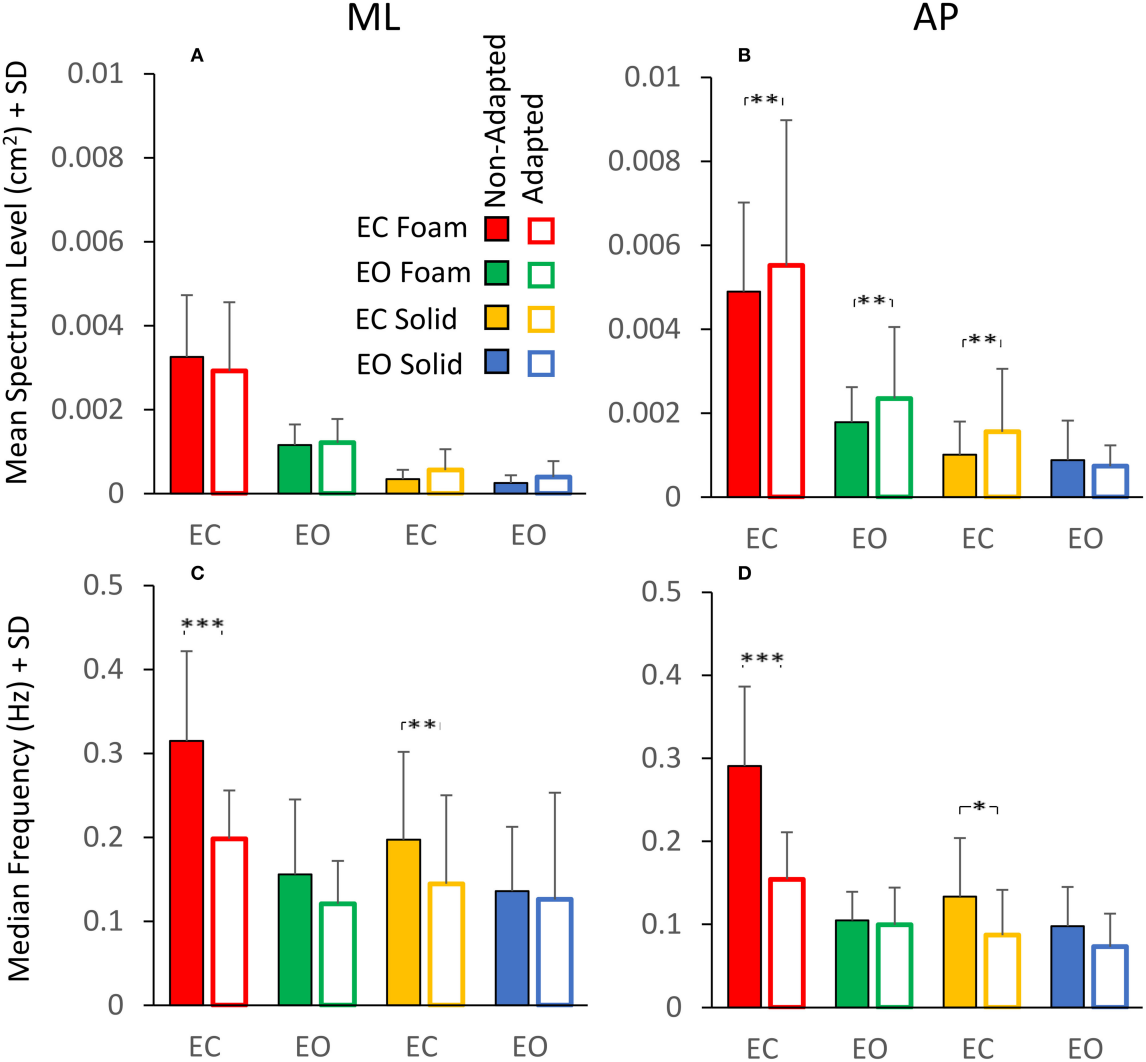
Effect of adaptation on mean Spectrum Level (upper panels) and Median Frequency (lower panels). In the ML direction **(A)**, the mean Spectrum Level is similar between the non-adapted (filled columns) and the adapted trials (empty columns). In the AP direction **(B)**, the mean Spectrum Level increases with adaptation, except for the EO Solid condition. The Median Frequency **(C,D)** moves towards smaller values with adaptation, particularly with EC, for both Foam and Solid supports (red and yellow, respectively). Asterisks indicate significant differences (*, *p* < 0.05; **, *p* < 0.01; ***, *p* < 0.001).

The analysis of the changes in sway induced by the adaptation gave an additional illustration of the insecure relation between Spectrum Level and Median Frequency. Figure 7 shows a summary of the findings relevant to the spectrum and the frequency of sway in the four conditions tested. Figures 7A,B show the mean Spectrum Level for the non-adapted (filled bars) and the adapted trials (empty bars) under both visual and both support conditions in the ML and AP directions. Rm ANOVA showed a nearly significant difference in the Spectrum Level between the non-adapted and the adapted trials for ML and AP data combined [main effect, *F*_(1, 19)_ = 3.9, *p* = 0.06], and the interaction between the non-adapted and adapted trials, and the ML and AP directions were significant [*F*_(1, 19)_ = 4.67, *p* < 0.05, $\eta_{\text{p}}^{2}$ = 0.19]. In fact, the Spectrum Level increased in the AP direction with adaptation, and the difference between the non-adapted and adapted trials was significant for all conditions (*post-hoc, p* < 0.01 for all comparisons), except EO Solid (*p* = 0.4).

Figures 7C,D show that the Median Frequency of the spectrum diminished with the adaptation [main effect, *F*_(1, 19)_ = 35.3, *p* < 0.001, $\eta_{\text{p}}^{2}$ = 0.99]. There was no interaction between the non-adapted and adapted trials and the ML and AP directions, because this effect was common to both directions. The interaction between non-adapted and adapted trials, and Foam and Solid support [*F*_(1, 19)_ = 6.11, *p* < 0.05, $\eta_{\text{p}}^{2}$ = 0.65] and the interaction between the non-adapted and adapted trials and visual conditions [*F*_(1, 19)_ = 18.3, *p* < 0.001, $\eta_{\text{p}}^{2}$ = 0.98] were significant. With EC, the Median Frequency diminished both with Foam (*post-hoc, p* < 0.001) and Solid (*p* < 0.05) support for both ML and AP directions. With EO, the difference in Median Frequency between non-adapted and adapted trials never reached significance for either base of support conditions or ML and AP directions (*p* > 0.06 for all comparisons). Comparison of Figure 7A with Figure 7C and of Figure 7B with Figure 7D shows the contrast between Spectrum Level and Median Frequency changes. Whilst the latter diminished in all conditions, the Spectrum Level remained approximately constant between non-adapted and adapted trials or even increased in AP direction (see the first three columns in Figure 7B).

**Figure 8:**
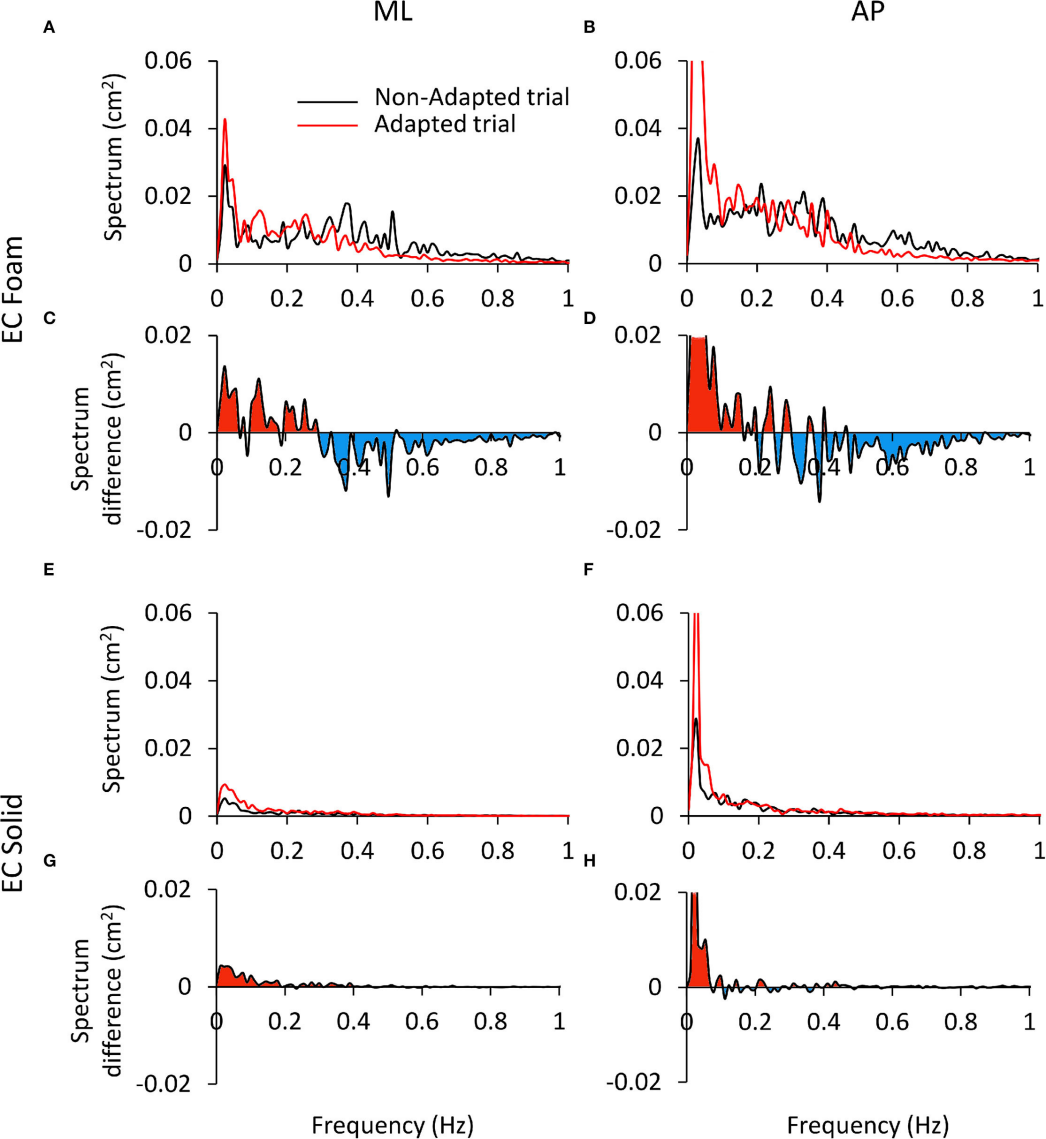
Differences in the power spectrum between the non-adapted and adapted trials. **(A–D)** refer to EC Foam, **(E–H)** to EC Solid condition. In **(A,B,E,F)** the mean Spectrum profiles of the non-adapted (black) and adapted trials (red) are reported for ML (A and E) and AP directions **(B,F)**. In **(C,D,G,H)**, the difference (adapted minus non-adapted) between the spectrum profiles of the non-adapted and adapted trials is shown. Red areas indicate a larger amplitude of the spectrum in the adapted than the non-adapted trial, oppositely the blue areas. With adaptation, the Spectrum Level increases at low frequencies (<0.3 Hz) and decreases at high frequencies (>0.3 Hz). This effect is limited to the low frequency range on a Solid support. The ordinates are capped in all the panels.

The reason for these incongruences is offered by inspection of the plots of the profiles of the spectra (ML and AP) of the non-adapted and adapted CoP excursions in Figure 8. This shows the mean frequency spectra for the EC Foam (Figures 8A–D) and EC Solid (Figures 8E–H) conditions (ML and AP). The superimposed traces refer to the non-adapted (the black line) and the adapted trials (the red line) on Foam (Figures 8A,B) and on Solid surfaces (Figures 8E,F). Clearly, adaptation produced a decrease in the Median Frequency. The mean Spectrum Level could change, depending on the extent of the concurrent decrease or increase of the modified values at low or high frequencies. Figures 8C,D show the difference between the mean spectrum profiles of the adapted and the non-adapted stance trials. The coloured areas indicate the frequency range where the spectrum level increased (red) or decreased (blue) with adaptation. For the EC Foam condition, the amplitude of the lower frequencies (< about 0.3 Hz) increased in the adapted trials, whereas the amplitude of the higher frequencies (>about 0.3–0.4 Hz) diminished.

A dividing point in the abscissa could be identified around 0.3 Hz for both ML and AP directions, with some disarray around that frequency in the AP direction. Hence, on the whole, the mean Spectrum Level did not change much with adaptation, owing to sort of “netting” between frequencies. Figures 8E–H shows the differences in the spectrum levels for the condition EC Solid. These changes were smaller than for EC Foam and limited to the very low frequencies in keeping with the minor effects of adaptation in this more ‘stationary’ condition.

A post-adaptation regression of squared SD vs. Spectrum Level was drawn. In the non-adapted much as in the adapted trials, the relationship between squared SDs and mean Spectrum Levels identified a straight line in all visual and surface conditions and in both ML and AP directions. As shown in Table 6, the slopes of the regression lines (i.e., the angular coefficient of 180) and the determination coefficients (close to 1) were the same as for the non-adapted trials illustrated above in Figure 4. This was true regardless of the adapted trials featuring a shift to smaller Median Frequency values and to larger Spectrum Levels in AP than in ML direction compared to the non-adapted trials.

**Table 6:** Relationship between CoP SD^2^ and mean Spectrum Level in the adapted trial.

**Direction**	**Condition**	**Equation**	***R^**2**^***
ML	EC foam	y = 181.1 x + 0.0009	0.99
	EO foam	y = 180.9 x + 0.0002	0.99
	EC solid	y = 180.0 x + 0.0004	1
	EO solid	y = 180.5 x - 0.0005	0.99
AP	EC foam	y = 180.6 x + 0.0064	0.99
	EO foam	y = 180.2 x + 0.0015	1
	EC solid	y = 180.1 x + 0.0008	1
	EO solid	y = 180.8 x - 0.0004	0.99

**Figure 9:**
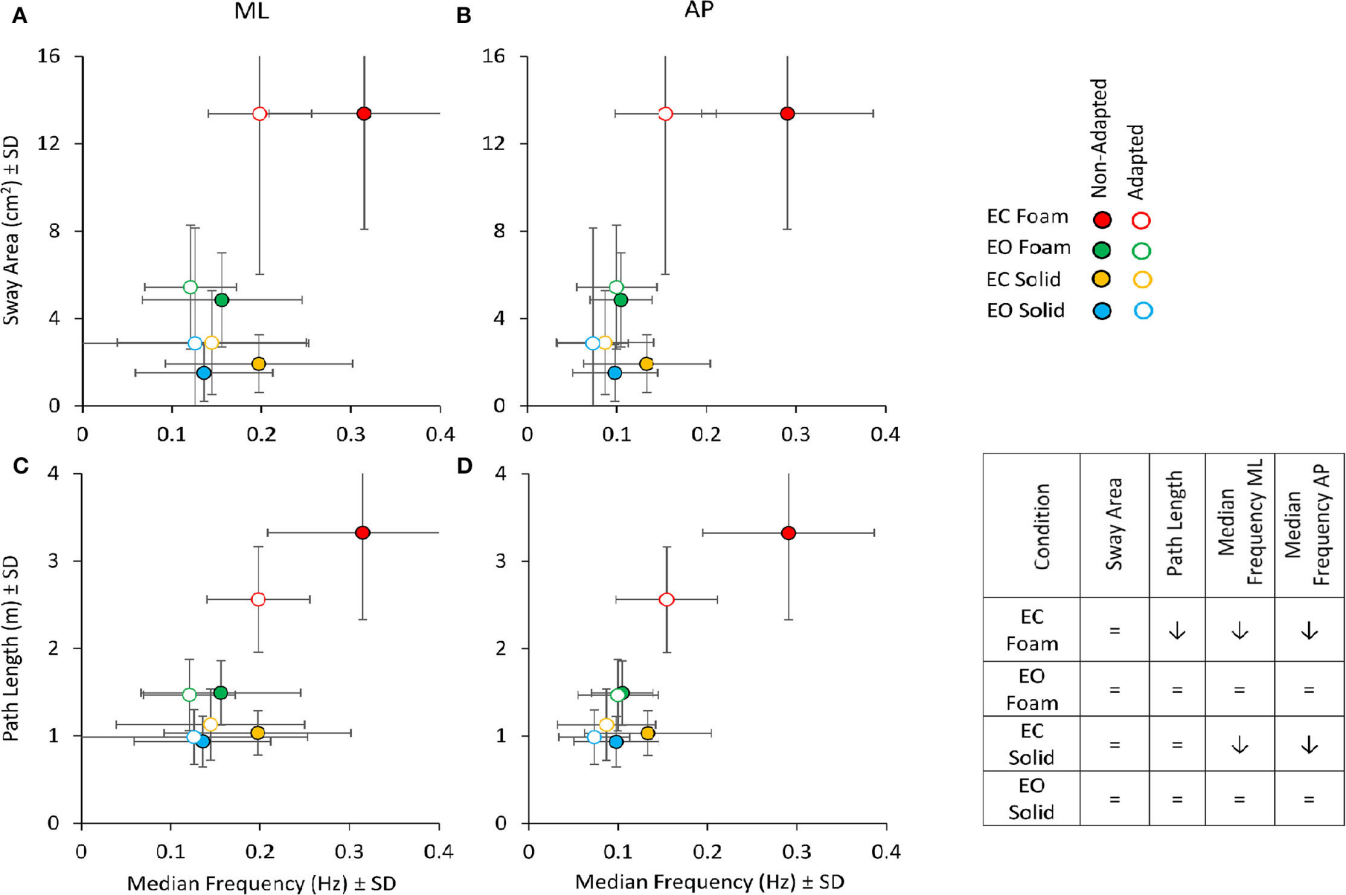
Sway Area (top panels) and Path Length (bottom panels) vs. Median Frequency for non-adapted (filled symbols) and adapted trials (empty symbols). For each visual condition and support surface, the mean value of Sway Area or Path Length is plotted against the corresponding mean value of the Median Frequency for both ML **(A,C)** and AP **(B,D)** directions. Note that the values of Sway Area and Path Length are the same for the left and right panels. Filled symbols refer to the non-adapted, open symbols to the adapted trial. With adaptation, both Path Length and Median Frequency decrease, especially in the EC Foam condition. Sway Area, except for the EC Foam, tended to increase with adaptation, even if not significantly. The Table inset summarises the effect of adaptation on the considered variables (= indicates no significant effect of adaptation; ↓ points to a significant diminution in the value of the variable).

The relationships between Sway Area or Path Length with the Median Frequency are summarised in Figure 9 for the ML (Figures 9A,C) and AP directions (Figures 9B,D), considering the average values of the non-adapted (filled symbols) and the adapted trials (open symbols). Adaptation slightly increased the values of Sway Area (in all conditions, except EC Foam, where Sway Area was always the largest) in the presence of a systematic diminution of the Median Frequency (both in ML and AP directions). This was true for both visual conditions and both supports, where the open symbols (adapted trials) were always displaced upwards and leftwards. For Path Length, however, the reduction due to adaptation was clear only in the EC Foam condition (technically, there are some increases, albeit not significant, for the adapted trials in Solid condition, where blue and yellow empty circles appear a little above the filled ones).

Rm ANOVA showed no main effect of adaptation on Sway Area [*F*_(1, 19)_ = 1.54, *p* = 0.23]. As expected, there was a difference between Foam and Solid supports [main effect, *F*_(1, 19)_ = 113.12, *p* < 0.001, $\eta_{\text{p}}^{2}$ = 0.85] and between visual conditions [main effect, *F*_(1, 19)_ = 715.3, *p* < 0.001, $\eta_{\text{p}}^{2}$ = 0.79]. Conversely, for Path Length (Figures 9C,D), an effect of adaptation was obvious (adapted < non-adapted) [*F*_(1, 19)_ = 10.9, *p* < 0.01, $\eta_{\text{p}}^{2}$ = 0.36]. There was also a difference between Foam and Solid supports [main effect, *F*_(1, 19)_ = 160.6, *p* < 0.001, $\eta_{\text{p}}^{2}$ = 0.89] and between visual conditions [main effect, *F*_(1, 19)_ = 100.2, *p* < 0.001, $\eta_{\text{p}}^{2}$ = 0.84]. An effect of adaptation on Path Length was clear only in the EC Foam condition (*post-hoc, p* < 0.001), whereas, in the other three conditions, Path Length values were small and unaffected by adaptation (*p* > 0.3 for all comparisons).

### Differences Across Variables Show Up When the Effects of Vision, Support Surface or Adaptation Are Expressed by the Romberg Quotient (EC/EO)

**Figure 10:**
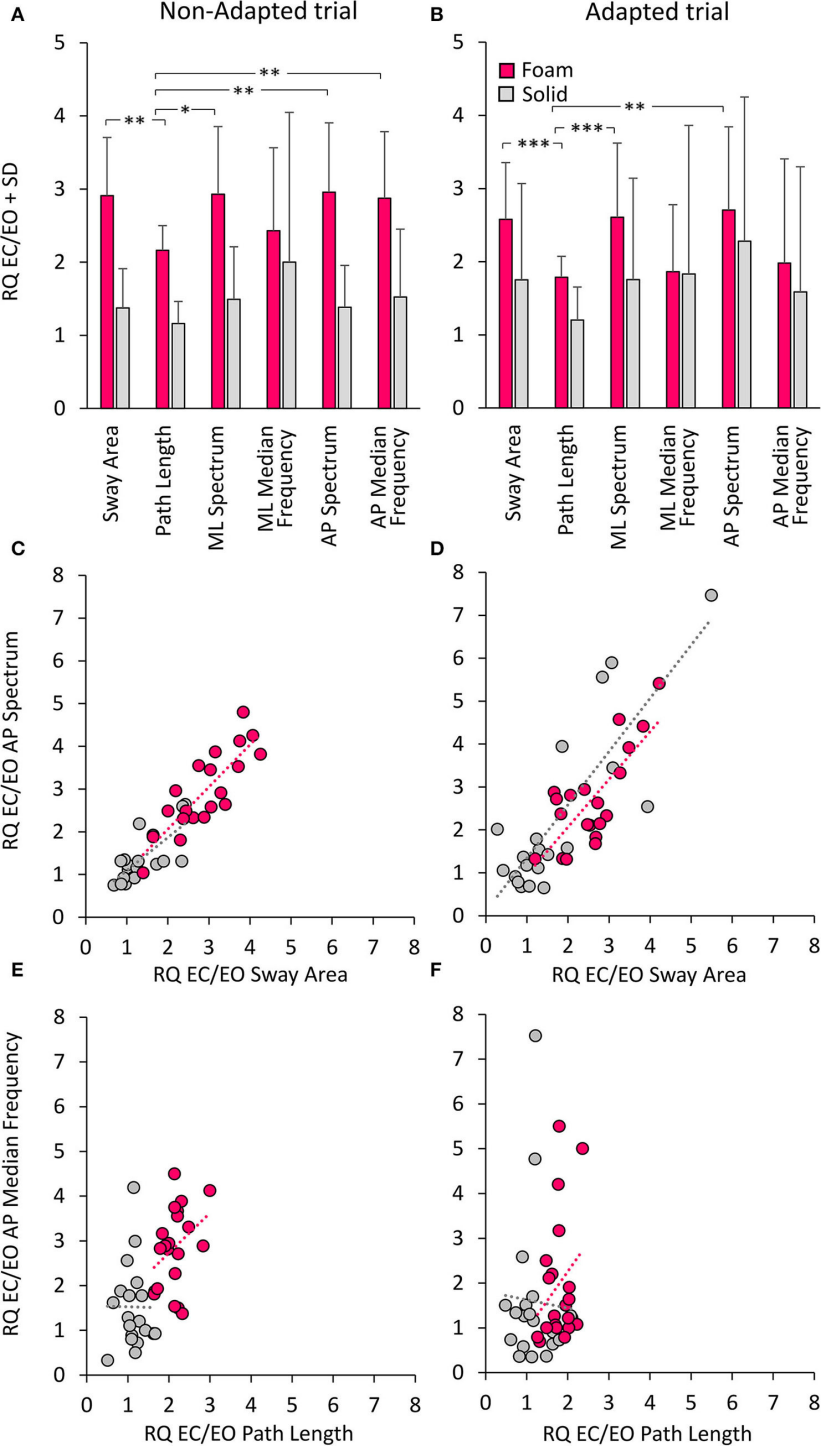
Romberg Quotients (RQ) of geometrical and spectral variables. The top panels show the RQs for the non-adapted **(A)** and adapted **(B)** trials and for the Foam (pink) and Solid (grey) support conditions. Asterisks indicate significant differences (*, *p* < 0.05; **, *p* < 0.01; ***, *p* < 0.001). The middle panels show the relationship between RQs of AP Spectrum Level and RQs of Sway Area for both non-adapted **(C)** and adapted trials **(D)** (Foam, pink; Solid, grey). Each circle corresponds to a subject. The bottom panels show the relationship between RQs of AP Median Frequency and RQs of Path Length for both non-adapted **(E)** and adapted trials **(F)**. The apparent proportionality between the RQs calculated on Median Frequency and Path Length is related to the overall shift to the left of the Path Length dots in Solid support condition.

Figure 10 shows the Romberg Quotients (RQ) for the geometrical and spectral variables in selected conditions. Figures 10A,B show the RQ of Sway Area, Path Length, Spectrum Level, and Median Frequency for the ML and AP directions, calculated for the non-adapted and the adapted trials. Friedman's ANOVA showed a difference in the RQs of the considered variables [main effect, χ^2^ (5 df) = 13.8, *p* < 0.05]. For all variables pooled, there was no difference in RQ between the non-adapted and the adapted trials [main effect, χ^2^ (1 df) = 2.13, *p* = 0.14]. The comparison between the pink and grey bars in Figures 10A,B shows that the RQs were always larger for all variables when standing on the Foam (pink bars) than on the Solid support [main effect, χ^2^ (1 df) = 62.6, *p* < 0.001].

In the non-adapted trials on Foam, the Path Length RQ was different from that of Sway Area and of mean ML and AP Spectrum Level (Wilcoxon's *post-hoc, p* < 0.05 for both comparisons). A similar pattern was observed also in the adapted trials, where RQ was smaller for Path Length than Sway Area (*p* < 0.001) and mean Spectrum Level (*p* < 0.01 for both ML and AP directions). On Solid support, there were no differences in the RQs between Sway Area, Path Length or Spectrum Level, either in ML or AP directions (*post-hoc, p* > 0.1 for all comparisons). The RQ of Path Length was not different from that of Spectrum Level on Solid support both in non-adapted and adapted trials (*p* > 0.17 for both ML and AP directions). The RQ of Median Frequency (both ML and AP) was very similar to that of Sway Area and Spectrum Level, both on Foam and Solid support and both in non-adapted and adapted trials (*p* > 0.11 for all comparisons). Overall, looking at both the non-adapted and adapted trials on Foam, the most conspicuous result was the similarity of the RQs for Sway Area and Spectrum Level on the one hand (*p* > 0.08 for all comparisons), and that between Path Length and Median Frequency for the adapted trials on the other (*p* > 0.8 for all comparisons).

Figures 10C,D show the concurrent changes in the RQs for the Spectrum Level in the AP direction and Sway Area in the non-adapted and adapted trials, respectively, on Foam (pink circles) and Solid support (grey circles). Similar plots were obtained for the ML direction. Across subjects, support conditions, and non-adapted or adapted trials, a strong relationship between the RQs of Spectrum and Sway Area was observed. Table 7 shows that the slope of the regression line (close to the identity) and the determination coefficients (close to 1) were comparable between Foam and Solid support, and were, in turn, similar to the values of the regression line fit to the data points of both Solid and Foam collapsed (non-adapted: y = 0.98x + 0.07, *R*^2^= 0.82, *p* < 0.001; adapted: y = 1.11 x + 0.08, *R*^2^= 0.63, *p* < 0.001). Such correspondence was not present for the relationship between Median Frequency and Path Length (Table 7). Adaptation reduced the RQ of Path Length, but not that of Median Frequency to the same extent. In this case, the regression fitted to the pooled data (non-adapted: y = 1.1 x + 0.36, *R*^2^ = 0.34, *p* < 0.001; adapted: y = 0.41 x + 1.17, *R*^2^ = 0.01, *p* = 0.44) points to the substantial invariance of the Romberg Quotients of Path Length in spite of a large scatter of those of the Median Frequency. This was true for both the non-adapted and the adapted data sets.

**Table 7:** Relationship between RQs of Sway Area and AP Spectrum Level and between RQs of Path Length and AP Median Frequency in the non-adapted and adapted trials.

**RQ**	**Condition**		**Equation**	***R^**2**^***	**Slope different from zero**	**Foam vs. Solid**
Sway area vs. AP spectrum level	Non-adapted	Foam	y = 0.98 x + 0.08	0.69	*p* < 0.001	*p* = 0.54
		Solid	y = 0.77 x + 0.32	0.52	*p* < 0.001	
	Adapted	Foam	y = 1.11 x - 0.16	0.58	*p* < 0.001	*p* = 0.22
		Solid	y = 1.24 x + 0.11	0.69	*p* < 0.001	
Path length vs. AP median frequency	Non-adapted	Foam	y = 0.89 x + 0.94	0.11	*p* = 0.15	*p* = 0.19
		Solid	y = −0.03 x + 1.55	0.00007	*p* = 0.97	
	Adapted	Foam	y = 1.30 x - 0.35	0.07	*p* = 0.26	*p* = 0.57
		Solid	y = −0.18 x + 1.81	0.002	*p* = 0.84	

## Discussion

This investigation is part of the continuous effort in search for markers of postural control, here assumed to be attainable by examination and analysis of the centre of feet pressure excursions over time (46, 59, 63–67). We hypothesised the existence of possible non-marginal incongruities between the geometric properties of body sway (Sway Area and Path Length) and the Standard Deviation (SD) of the CoP excursions along ML and AP directions. Furthermore, we aimed to contrast the results of the spectral analysis of the CoP excursions (mean Spectrum Level and Median Frequency) with Sway Area and Path Length. This issue has been addressed here by reanalysing from an unconventional perspective the body sway data obtained during a recent investigation on the adaptation of stance to repeated trials in a population of young, healthy volunteers (1). The necessary data variability has been offered by two support surface conditions (Foam or Solid), two visual conditions (eyes open, EO; or eyes closed, EC) and the adapted conditions in the same population of healthy young subjects. By leveraging the changes in geometric measures and in the spectral data produced by the analysis of the different experimental conditions, we addressed here the distinct relationships across these variables and the effects on the postural control mode related to the visual, support surface and adapted conditions, also in the view of identifying any potential relevance to clinical practise.

We have confirmed the stabilising effects of the vision and solid base of support on the CoP excursions during standing without additional constraints beyond keeping equilibrium in a spontaneous attitude (e.g., there was no instruction to “stand as still as possible”). When vision was occluded, or a compliant foam support was used, or both, all geometric and spectral measures underwent major and significant changes, but with notable departures from a simple scheme of concurrent increase as a function of the hypothesised unsafe stance condition (e.g., EC or Foam support) (1, 68) or of concurrent decrease with adaptation.

### The Loose Correspondence Between Sway Area and Path Length

Sway Area estimates the size of the surface covered by the excursion of the CoP during the test, and the Path Length is the total sum of all the CoP displacements from one to the next sampled position. Both measures are influenced by the sum of all the inertial and voluntary forces acting onto the force platform. However, it is easy to figure out that the two measures do not necessarily correspond. In the scatterplot of Sway Area vs. Path Length, the data points of all the subjects have very low values for both variables with EO Solid, larger values with EC Solid, still larger with EO Foam, and very large and more variable values with EC Foam. Furthermore, no or weak relations between these variables were present when standing on the Solid support, whereas significant relationships occurred when standing on Foam, regardless of the visual condition. This is in keeping with the findings of several previous investigations, even if those studies did not always mutually compare these variables across distinct sensory and surface conditions in the same population (69–71).

Seen from a different point of view, Sway Area has a tendency to be very small in the EO Solid condition, whilst Path Length can have substantial values. These can be similar to those observed when Sway Area is larger, as in the EC Solid or EO Foam condition. This is not surprising, because the CoP excursions can have some length but be packed in a very small space, in particular when the frequency of the small excursions is high. In other words, Sway Area can be more sensitive to the large excursions towards the limits of stability (72), whilst Path Length can give a better indication of the presence of a ‘stiffening’ strategy (51), featuring fast back-and-forth displacements of the CoP. An intermittent control of quiet upright standing (73–76) would likely favour increase in Sway Area rather than Path Length. When the conditions are unstable (as in EC Foam) and the subjects search for a safe posture, the association between Sway Area and Path Length (as assessed by the determination coefficient) improves but remains moderate, being probably dependent on the idiosyncratic behaviour of the single participants (77). All in all, the measures of Sway Area and Path Length cannot be considered equivalent, or redundant.

### Sway Area Bears a Better Association Than Path Length With the SD Variance of ML and AP Excursions of the CoP

In the common and easy standing posture as that exploited here (parallel feet at pelvis width), the values of Sway Area and Path Length are largely proportional to the values of the SDs of the CoP excursions in both the ML and the AP directions. When the Sway Area and Path Length values are plotted against the squared SD (the variance of the time series of the CoP excursions), a linear regression was found, and the slopes of the straight lines become very similar across conditions. Therefore, the variance of the CoP excursion along the ML and AP directions is an exact predictor of the Sway Area and Path Length. Anyhow, the squared SD is better associated with Sway Area than Path Length (the coefficients of determination are always >0.75 and always <0.60 in Sway Area and Path Length, respectively).

### The Squared SD Corresponds to the Mean Level of the Spectrum

The squared SD of the CoP and the mean Spectrum Level are both higher with Foam than Solid support, higher with EC than with EO and higher for AP than for the ML direction. These correspondences are not surprising (8). As expected from the structure of the formulae for the calculation of the SD and of the Spectrum in the ML and AP directions, the relationships between squared SD and mean Spectrum Level identify a straight line with a zero intercept and a slope of 180. This exact number depends on the frequency of sampling and the time duration of the acquisition. Of note, this coefficient is the same for all four standing conditions.

Therefore, the overall amplitudes of the oscillations of the CoP in the two directions of the horizontal plane are accurately determined, except for a coefficient set by the sampling frequency and trial duration, by the SD values of their zeroed-mean trace. In a sense, the Spectrum Level becomes a redundant and useless marker once the variances of the time series of the CoP are available. This statement is true regardless of the base of support and sensory conditions, and, therefore, regardless of the postural control strategies and whatever idiosyncratic characteristics of the participants may confound the measures of Sway Area and Path Length. In other studies (78, 79), the result of the frequency analysis of the CoP signal is reported as Power Spectral Density (PSD). The PSD represents the frequency distribution of the power associated with a signal, where the squared rms amplitude of the spectrum is divided by the frequency resolution (0.011 Hz in our study). Thereby, the mean Spectrum Level calculated on the PSD would be equal to the mean Spectrum Level calculated on the power spectrum divided by the frequency resolution. Hence, the relation between the squared SD and the PSD would be again a straight line passing through the origin of the graph, where the regression coefficient changes from 180 to 2 (i.e., 180^*^frequency resolution, if the frequency resolution is 0.011). No wonder that, when considering the data from all subjects, an imperfect but convincing correspondence appears between Sway Area and mean Spectrum Level, both being strongly related to the SD of the CoP excursions. In this light, both measures can be considered reliable markers of the amplitude of body sway. The relationship Sway Area vs. Spectrum Level is affected by the direction of sway (ML or AP) to a very limited extent (Table 3).

### The Postural Control May Not Be Fully Expressed by the Median Frequency of the Spectrum

The spectral analysis gives another major information on the characteristics of the CoP excursion along the ML and AP directions, i.e., the amplitude of the oscillation frequencies during the periods of interest. The Median Frequency depends on the distribution of the frequencies in the spectrum. Low frequencies are normally prevalent [e.g., (78, 80)], with a progressive decrease in amplitude from the low to the high frequencies. High frequencies have been described for the human body sway, dependent on the way people stand on the force platform [e.g., (51)], on body or limb tremors (featuring frequencies > 2.0 Hz) (81), and on the filtering and cut-off frequency of the signal. These Median Frequencies do change with the sensory and support conditions, as well as with adaptation (see below). Furthermore, Median Frequency is slightly higher for ML than AP directions. The relationship between Median Frequency and Spectrum Level is not simple and not easily described by one function, as shown by inspection of Figure 6. Beyond a very loose association, whereby all the data combined seem to be showing a mediocre proportionality, equal Spectrum Levels correspond to largely distinct Median Frequencies, and *vice versa*. For instance, the reshaping of the frequency spectrum by vision (EO) reduces the Median Frequency on Foam by about half or less of its value EC, and by only about 30% on Solid support (for Median Frequencies smaller than on Foam, see Figures 6A,B in the Results Section). The small effect of vision manipulation on Median Frequency has been described already in Sozzi et al. (51) on Foam and Solid supports (no adaptation), and is confirmed here. Similarly, the use of a Solid support overall reduces the Median Frequency under both vision conditions. These significant adjustments occur for the oscillations in both the ML and the AP directions.

However, the values of the Median Frequency bear a substantial overlapping of the individual values across conditions (Figures 6C,D). Hence, except for the condition EC on Foam, where the Median Frequency reaches the greatest values (from as low as 0.1 Hz up to 0.5 Hz), Median Frequency does not attest to the standing conditions at hand (54, 82–84). Duarte and Zatsiorsky (85) computed higher median frequencies in young adults standing on Solid support, and compared their finding with that of other studies. It should be mentioned that we high-pass filtered the signal at 0.01 Hz instead of 0.05 Hz as in Duarte and Zatsiorski (85), thereby favouring the appearance of lower Median Frequency values. Others have found that low frequencies are decreased whilst high frequencies are increased by height exposure (real and virtual) (86, 87). Trial duration (88), signal recording and processing, and the modest participant numbers may also partly explain the discrepancies. In a sense, these discrepancies suggest caution in interpreting the values of the Median Frequency, which have been previously suggested to be sensitive indexes, for instance, of healthy ageing (12) and calf muscle fatigue (89). All in all, it seems that Median Frequency of sway *per se* is a poor predictor of the amplitude of the CoP excursions and cannot tell much about the magnitude of the oscillation pattern of the body sway, either in the ML or AP direction.

### Changes in the Postural Control Strategy Are Disclosed by Changes in the Median Frequency Induced by Adaptation

Adaptation to repeated stance trials (not to be confused with accommodation to some imposed visual or physical perturbations of posture) produces selective changes in sway variables (1). The mean Spectrum Level shows minor changes between the non-adapted (the first trial) and the adapted trial on either Foam or Solid surface in the ML direction. In the AP direction, more consistent although limited increases in the mean Spectrum Level are observed in all adapted conditions, except in the most stable condition (EO Solid).

Moreover, across the subjects, there was no significant regression between Spectrum Level and Median Frequency for all conditions, except for EC Foam in AP direction. The decrease in Median Frequency with adaptation in the EC conditions (both Foam and Solid support), in the absence of major changes in the mean Spectrum Level, must be traced back to the shift in the spectrum profile from the high to the low frequencies (90). The decrease in Median Frequency is much larger for the EC Foam condition but is also present in the other conditions even if of lesser magnitude (Figure 7). This decrease does not affect Sway Area but is accompanied by a reduction in Path Length, which is obvious for EC Foam and negligible for the other conditions (Figure 9). Others have already shown that prolonged balance training reduces Path Length but not Sway Area (49), and this effect was again significant without vision on Foam.

The Median Frequency can have similar values in the adapted EC Foam and adapted EC Solid conditions (e.g., in the AP direction) or in the EO Foam and EC Solid conditions (in the ML direction), but body sway is different between visual and support conditions. Furthermore, the Median Frequency may not be a predictor of Sway Area, because Sway Area is very similar in the adapted and non-adapted trials, in spite of changes in the Median Frequency. This discrepancy is not surprising. If we admit that the adaptation process aims to improve balance at the same time that it decreases the effort for controlling the upright stance, in keeping with the notion put forward by Kiemel et al. (91) (stabilising upright stance with near-minimum muscle activation), a large Sway Area may not necessarily correspond to instability. The discordance between Sway Area and Median Frequency under the most challenging condition confirms that a stabilising process had taken place with adaptation, but it does not necessarily imply reduction of the total surface of the wandering CoP. We suggest that the increase in low-range frequencies with repetition on Foam is explained by subjects' relaxation, implying diminished muscle stiffness and reduced high-frequency “trembling” (1, 92). In turn, the reduction in mid-range frequencies shows that less effort is sufficient for controlling body position whilst maintaining allowance for exploration and feedback.

Jeka et al. (93) suggested that balance control is under the influence of body's velocity information rather than position or acceleration. Since the Median Frequency is broadly proportional to the CoP velocity, the reduction in the Median Frequency with adaptation in the case EC Foam is a measure of progressive decrease in CoP velocity. The reduction in Median Frequency around 0.5 Hz would diminish the contribution of the vestibular control of sway (94). The absence of adaptation effects during stance on a firm surface, where the vestibular input may not be relevant, is, in turn, in keeping with the idea that proprioceptive cues dominate the control on solid grounds (95). On the other hand, the effect of adaptation is selective, because the Median Frequency *increases* under a different condition, i.e., fatigue induced by an intense treadmill exercise (96). Interestingly, fatigue induces co-contraction of antagonist leg muscles and greater articular stiffness, with increase in Median Frequency due to augmented oscillation frequencies > 0.5 Hz (97).

### Rambling and Trembling

An ample range in the CoP oscillation frequency may not be an odd or abnormal occurrence, since CoP not only measures the balance-correcting activities of the muscles but the passive motion of the body indirectly produced by those activities as well. In turn, the passive body motion is braked by ensuing muscle contractions and so on. No wonder that a healthy nervous system aims to reduce in strength and frequency the active correcting bursts and let the body passively oscillate whenever this is not threatening equilibrium. A reduction of Median Frequency in the adapted trials occurs when vision is occluded. This reduction can be attributed to a change in the postural control mode from the trembling to the rambling strategy (1, 5, 98). We show here that there is a frequency (~0.3 Hz) in the spectrum of the CoP oscillation that is unaffected by adaptation, as if those frequencies were hardwired and not amenable to changes in control. On the other hand, and importantly so, the process of adaptation leads to increase in the lower frequencies in the “rambling” range, and to a reduction of the higher frequencies by diminishing the “trembling” component of the spectrum (5, 99, 100).

Even if comparison is unwarranted and cannot be quantified, owing to the distinct sensory conditions (EO, EC, Foam, Solid) and the time effect (adaptation), we suggest that the adaptation processes underpinning these states may be, in part, shared. Adaptation reduces the Median Frequency by about one-third to one half (EC Foam, in ML and AP directions, respectively), whereas opening the eyes reduces the Median Frequency by one half and one third with Foam and Solid support, respectively. It seems that both vision and adaptation favour the operation of supraspinal contribution (pointed out by the increase in the low frequencies), where the visuo-vestibular integration would play a role (101). This can be contrasted with the increase in frequency noted in vestibular dysfunctions (79). At the same time, under conditions of reduced stability, adaptation reduces the excitability of reflex pathways (102) and the contribution of muscle stiffness (1). Beyond this process, adaptation further favours the appearance of very-low frequencies, or drifts (30, 103) with EC with respect to EO (1) and both with Foam and Solid support. Components of the CoP excursion at frequencies <0.05–0.1 Hz are more likely to occur both when visual feedback is prevented and when adaptation emerges (1, 51). These drifts increase the mean Spectrum Level in the low-frequency range and Sway Area at the same time that they decrease Median Frequency, as mentioned above.

This finding contrasts the process of adaptation to that of “automaticity” of postural control (104). In the former case, supraspinal control would prevail; in the latter, there would be a shift towards a greater contribution from higher oscillation frequencies, as evidenced in cognitive task conditions (105). It is easy to imply that a cognitive effort benefits from a “free” brain and exploits the brainstem-spinal centres for the control of stance, thereby promoting the appearance of higher frequencies in the spectrum. These changes in Median Frequencies are not accompanied by parallel changes in Sway Area.

### Ambiguous Information From the Romberg Quotient

The Romberg Quotient (RQ) quantifies the degree to which balance worsens when vision is removed and can be an index for identifying prospective fallers (106, 107). We found that, when standing on Foam, the effect of vision is plainly evident on the RQ. For all variables, the RQ can reach high values in some subjects, similar to those attained with a reduced base of support (108–110). The RQ is larger for Sway Area, and such a large value is common to the Spectrum Level, in both the ML and AP directions. Contrariwise, although large, the RQ of Path Length is significantly smaller than that of Sway Area, and similar to that of Median Frequency. This indicates that the RQ can be quite different, depending on the variable considered. On a Solid base of support, the reliability of the RQ would be questionable, since, on average, it is not different across variables.

It has been posited that, in abnormal conditions of higher brain centres, Sway Area diminishes on closing the eyes, whereas Path Length increases by a stiffening effort (111). Such divergence would be a marker of abnormal compensation, probably obtained by rigidifying the body. On the other hand, Kalron (112) showed that severely affected patients with multiple sclerosis may have an elevated Romberg Quotient, calculated on Sway Area data, and that this relates to poor walking and balance abilities. A parallel consideration of the Median Frequency of the spectrum might help identify the mechanisms of such behaviour and make sense of RQ in ageing (98, 113, 114) and disease states. Hence, any use of the RQ in the clinic would be valid under given and postulated conditions, and should not be taken as a general all-inclusive tool for estimating the role of vision in stance, less so when the characteristics of the support surface are left unnoticed. As an afterword, one should add that the mean RQ, obtained from the individual ratios of all the participants, does not exactly correspond to the RQs of the mean values of the considered measures. This is so because a particular ratio depends on the quite variable values of the sway measures. Briefly, in the case of accidentally very small Sway Areas values in an EO trial accompanied by very large Sway Area in a corresponding EC trial, the RQ would be exceedingly large. The difference between the RQs obtained from the individual ratios of all the subjects and the RQs calculated on the mean values of the variables proved to be not disproportionate, being on average (all variables pooled), about 13% (the former larger than the latter). Yet, the question lingers of whether it is more appropriate for the purposes of clinical investigation to employ functional tests, such as standing on one leg (19) rather than sway measurements on a force platform, more so on a rigid support, where reactive balance control may not be obvious (115).

### Proprioception and Vision on Firm and Compliant Support

On Foam compared to Solid support, there is a rapid motion of several joints, and, thus, the large proprioceptive barrage contributes to enhancing the sensory input and the postural muscle activity. Standing on Foam should not imply impaired control by inordinate or inaccurate use of the information from the active and passively moving joints and muscles, which is normally considered an axiom [e.g., (7, 116, 117)] that could be tenable only after fatigue (96). Any changes in muscle length, more so when rapid, activate, in fact, the large spindle afferent fibres, highly sensitive to the velocity of muscle stretch rather than the slow muscle changes in length as when standing on a Solid support (118, 119). And the spindle input is certainly funnelled to the appropriate motor pools (120–123) and supra spinal centres (124, 125). In this light, a larger RQ on Foam compared to Solid support would be expression of the role played by vision in modulating the central integration of vestibular and somatosensory information (126, 127) rather than of the mere advantage connected with the presence of an external (visual) reference (128). Fransson et al. (83) and Patel et al. (54) also showed that the oscillation frequency increases with EC compared to EO when subjects stand on Foam. In the adapted conditions, the effect of vision is again obvious when standing on Foam, but the related RQ values are much smaller than in the non-adapted conditions, suggesting progressive up-weighting of the proprioceptive information over time (1, 10, 129).

On a different vein, it should be kept in mind that the continuous motion of the joints largely reduces the passive joint stiffness by diminishing the short-range elastic component due to thixotropy (130). This would be all the more true for the muscles around the ankle joints standing on Foam (with increased sway) (131), suggesting that standing on foam could minimise the role of passive muscle-joint properties for the control of standing. Hence, standing on a compliant support would be challenging also because the reliance on (low) ankle stiffness must be compensated by the added, learnt modulation of the active neural control, to which vision would add weight.

### Limitations

The participants to these experiments were not instructed to stand “as still as possible” (28) under all test conditions. This would have been implausible on Foam, and it seemed illogical to ask them to employ a different attitude on a Solid surface. But this loose instruction likely enhanced the inter-individual variability and attenuated the statistical differences between measures.

We did not manipulate the between-feet distance (132) either, because this would have required a double number of trials, hardly accepted by many volunteers. But this study was not designed for highlighting the difference in CoP sway between the mediolateral and anteroposterior directions of the horizontal plane (109), which strongly depends on feet position (84).

The number of participants was limited, and the study was restricted to young healthy adults. This has been of minor consequence, because notable differences between postural measures could be pointed out anyway. However, this weakness prevents direct application to older people (16, 133) or people with balance problems (134–136). This is particularly annoying because comparison across cohorts requires identical methodological procedures and analytical tools.

Because we considered the spectrum within the frequency range up to 2.0 Hz, a range leaving out 2% of the total spectrum, we might have underestimated small but non-trivial activities in the higher frequencies. This would be a concern in the case the procedure would be applied to patients with motor disorders. Furthermore, we have not used here non-linear sway measures or compared the present variables with entropy measures, which have been proposed to assess the dynamics and complexity of the CoP displacements (137).

### Conclusions

Summing up, the main conclusions to be taken into account in view of practical applications are listed below:

1. The mean Spectrum Level and the SDs of the time series of the ML and AP excursions of the CoP convey the same concept. Their values also bear an acceptable correspondence with the Sway Area values. All together, these variables offer a safe indication of overall body sway and can be exploited as a gross measure of imbalance. However, Spectrum Levels and SD value depend on the feet position, inasmuch as both refer to the direction of sway (138–141).
2. Sway Area and Path Length cannot be considered equivalent measures. The former is an expression of overall postural “performance” (142), including the search mechanism to find the limits of stability (71), the latter of postural steadiness, including the effort to minimise sway (57). The discrepancies between Sway Area and Path Length are of minor concern on Solid support, but increase on Foam support, particularly with EC.
3. The RQs have largely different values between Foam and Solid supports, between non-adapted and adapted trials, and are greater when calculated on Sway Area or mean Spectrum Level than on Path Length and Median Frequency. Hence, they should be used with caution when selecting markers of instability connected with vision occlusion or vision impairment (143).
4. The above discrepancies are of minor concern on Solid support, but they widen on the Foam pad, particularly with EC. Ahmadi et al. (144) identified standing on Foam EC as the most discriminative condition for classifying neurological patients based on data mining and machine learning techniques. Most likely, standing on foam can relate to incidence of falls. Further studies are needed where this issue is focussed on appropriate cohorts and *ad hoc* analyses (145). However, falling rarely happens during stance in the absence of external perturbations (however small). Rajachandrakumar et al. (146) had individuals standing on a solid surface and applied perturbations to them to induce stepping. They found that scarce mediolateral CoP variability in young adults was related to increased odds of stepping. Adding spectral analysis data may help fine-tune the conclusions on the underpinning mechanism (147).
5. The effects of the changes induced by adaptation produce a degree of uncertainty for the interpretation of the body sway measures. This is an issue when the average of successive trials is taken to get more reliable values for the measurements. This seems to be relevant for Median Frequency and Path Length, less so for Sway Area and for Spectrum Level or SD in the ML and AP directions.
6. Overall, standing on Foam is clearly more telling than standing on a Solid support. Although it has been suggested that standing on Foam and Solid supports does not rely on qualitatively different postural controls (38, 148), a compliant support certainly highlights features that are not easily derived with a firm base of support (83, 117, 149).

The excursions of the CoP during upright stance are an indirect measure of a person's ability to maintain balance. As mentioned in the Introduction, there has been, over the years, a continuous search of simple markers of body sway appropriate for dissecting out with some confidence measures of postural control in different populations, varying for gender and age (150–154), health status (155, 156), and medical conditions (56, 72, 157, 158) under various vision and support surface settings (159, 160). The present analysis offers a rationale for exploiting the information yielded by the force platforms but calls for further investigations before providing easy markers of postural control in older subjects or patients (161) or as a consequence of administration of various drugs [e.g., (162–164)] or else of balance rehabilitation treatments (52, 165–168). Our understanding of the control of standing and its fragility would be likely improved by exploiting compliant surfaces. It is good that patients can easily be put on Foam (169–173), and the safety harness would not alter their behaviour (174).

The findings shed further light on the effect of vision and support surfaces (175) and of adaptation on the control of stance based on the analysis of geometric and spectral measures. The large variation in these measures due to individual characteristics (e.g., visual and kinaesthetic dependence, (176, 177), muscle, (178), control strategies (27, 58), age (179) asks for a parallel examination of different markers before firm conclusions can be drawn from the findings. However, as discussed above, there is a fair correspondence between distinct measures that can help resolve apparent incongruities. Overall, the findings suggest that indiscriminate functional or pathophysiological implications from a single measure or a set of a few different measures are not warranted. For instance, Median Frequency is least sensitive to increased levels of difficulty (180), while it is sensitive to adaptation to Foam and to successive standing trials so that one has to be aware of this when repeating tests (88, 181, 182).

## Data Availability Statement

The original contributions presented in the study are included in the article, further inquiries can be directed to the corresponding author.

## Ethics Statement

Participation was voluntary and the protocol was approved by the Local Review Board (Istituti Clinici Scientifici Maugeri SB, approval number #2564-CE). The participants provided their written informed consent to participate in this study.

## Author Contributions

MS conceived the idea for the study. SS performed the recruitment of the participants and the collection of data. SS and MS performed the data analysis and drafted the article. SG revised it critically for important intellectual content. All authors approved the submitted version.

## Funding

The funding for this study was provided by the ICS Maugeri SB through the Ricerca Corrente programme of the Italian Ministry of Health. The Ministry of Health, responsible for the Ricerca Corrente funding, was not involved in the study design, collection, analysis, interpretation of data, the writing of this article or the decision to submit it for publication.

## Conflict of Interest

SG is employed by Rsgbiogen. The remaining authors declare that the research was conducted in the absence of any commercial or financial relationships that could be construed as a potential conflict of interest.

## Publisher's Note

All claims expressed in this article are solely those of the authors and do not necessarily represent those of their affiliated organizations, or those of the publisher, the editors and the reviewers. Any product that may be evaluated in this article, or claim that may be made by its manufacturer, is not guaranteed or endorsed by the publisher.
